# Tianhuang formula attenuates cardiomyocyte pyroptosis in myocardial infarction by suppressing oxidative stress and the cGAS–STING–NLRP3 axis

**DOI:** 10.3389/fimmu.2026.1761299

**Published:** 2026-02-20

**Authors:** Meiling Yan, Yifan Chen, Guida Cai, Xi Zhang, Kunping Li, Duosheng Luo, Lexun Wang, Xianglu Rong, Jiao Guo

**Affiliations:** Key Laboratory of Glucolipid Metabolic Disorder, Ministry of Education of China Guangdong, Institute of Chinese Medicine, Guangdong Metabolic Diseases Research Center of Integrated Chinese and Western Medicine, Key Laboratory of Metabolic Disease Prevention and Treatment of Traditional Chinese Medicine, Guangdong Pharmaceutical University, Guangzhou, Guangdong, China

**Keywords:** cardiomyocyte pyroptosis, cGAS–STING pathway, immune-inflammatory response, myocardial infarction (MI), Tianhuang Formula (THF)

## Abstract

**Background:**

Myocardial infarction (MI) remains a leading cause of morbidity and mortality, driven by ischemia/reperfusion injury, excessive inflammation, and maladaptive ventricular remodeling. Although acute reperfusion strategies have improved short-term outcomes, effective interventions targeting post-infarction inflammation and structural deterioration remain limited. Tianhuang Formula (THF), a patented two-herb prescription traditionally used to promote circulation and alleviate stasis, has shown potential cardioprotective properties, yet its mechanisms in MI remain insufficiently defined.

**Aim of the study:**

To evaluate the therapeutic effects of THF in a mouse MI model induced by left anterior descending (LAD) coronary artery ligation and elucidate its underlying molecular mechanisms.

**Materials and methods:**

Echocardiography was performed at 3 and 28 days post-MI to assess cardiac function. Network pharmacology integrated with transcriptomic profiling identified pathways potentially targeted by THF. Western blotting, immunohistochemistry, primary cardiomyocyte assays, and molecular docking were used for mechanistic validation.

**Results:**

THF significantly improved cardiac function during both the acute (day 3) and remodeling (day 28) phases of MI and reduced circulating inflammatory cytokines. Mechanistic analyses showed that THF mitigated myocardial hypertrophy by suppressing oxidative stress and inhibiting activation of the cGAS–STING pathway, thereby preventing downstream NLRP3 inflammasome-mediated pyroptosis and inflammatory cytokine production. Docking results further demonstrated strong binding affinities of key THF components—berberine, coptisine, and palmatine—to human Keap1 and cGAS.

**Conclusions:**

THF exerts cardioprotective effects by reducing oxidative stress, modulating the cGAS–STING–NLRP3 axis, and inhibiting cardiomyocyte pyroptosis, supporting its traditional use and highlighting its potential as a therapeutic candidate for MI.

## Highlights

- This study is the first to demonstrate that Tianhuang Formula (THF) improves cardiac function after myocardial infarction.
- THF suppresses oxidative stress in cardiomyocytes.
- THF inhibits the cGAS–STING–NLRP3 pathway to alleviate cardiomyocyte pyroptosis.
- Key components of THF (Berberine, Coptisine, Palmatine) show strong binding affinities with Keap1 and cGAS.

## 1 Introduction

Cardiovascular diseases (CVDs) have emerged as a dominant contributor to global illness and death and has become a significant challenge to global public health. A recent statistical update report from the American Heart Association revealed that approximately 19 million people died from cardiovascular diseases globally in 2020 (1). MI is a major culprit contributing to CVD-related deaths, with its pathogenesis involving complex interplay of large-vessel occlusion, microvascular dysfunction, myocardial remodeling, inflammatory responses, and neurohormonal activation, which may ultimately culminate in heart failure (HF) (2). Therefore, it has become a major challenge in the cardiovascular field to find new effective drugs and drug targets to improve the treatment rate of MI.

Cyclic GMP-AMP synthase (cGAS) acts as a cytoplasmic DNA sensor that plays different roles in the different stages of the innate immune inflammatory response (3). DNA damage is an important inducer of inflammation and cellular injury that activates the cGAS-STING signaling pathway, leading to inflammatory damage in MI (3, 4). In the early stages of MI, cGAS binds to double-stranded DNA (dsDNA) released from apoptotic or necrotic cardiomyocytes and synthesizes cyclic guanosine monophosphate-adenosine (cyclic GMP-AMP, cGAMP) (5). cGAMP binds to STING and facilitates the recruitment of TANK-binding kinase 1 (TBK1), which in turn activates interferon regulatory factor 3 (IRF3) to promote the expression of inflammatory mediators (3).

As a distinct mode of programmed cell death, pyroptosis is defined by the induction of inflammatory signaling and immune activation. Emerging evidence indicates that DNA damage can induce pyroptosis via the cGAS-STING-IRF3 signaling axis, which transcriptionally upregulates NLRP3 expression (6, 7). An increasing number of pharmacological interventions have demonstrated cardioprotective effects on myocardial infarction by targeting and suppressing key signaling cascades involved in pyroptosis (8, 9). The NLRP3-inflammasome, belonging to the NOD-like receptor family and characterized by a pyrin domain, is a multi-protein complex located in the cytoplasm (10–12). Once the NLRP3 inflammasome is activated, it promotes the activation of caspase-1, which subsequently processes pro-IL-1β and pro-IL-18 into their mature, biologically active forms. Meanwhile, caspase-1 cleaves gasdermin D (GSDMD) to release its N-terminal fragment (N-GSDMD), which embeds into the plasma membrane to create pores. These membrane pores act as the key executors of pyroptosis, leading to cell lysis and initiating downstream inflammatory and pathological responses (13, 14).

Traditional Chinese Medicine (TCM), with its roots stretching back millennia, has evolved through continuous refinement and clinical application, forming a comprehensive medical system for the prevention and treatment of diverse diseases (15). THF is a patented TCM compound composed of *Panax notoginseng* (Burkill) F.H. Chen and *Coptis chinensis* Franch, with pharmacological effects of promoting blood circulation and resolving blood stasis. Its design is based on Professor Guo Jiao’s therapeutic theory of “Tiaogan Qishu Huazhuo” for glycolipid metabolic disease, aiming to improve cardiac blood flow, facilitate the clearance of metabolic waste, and optimize the myocardial metabolic environment (16–18). The scientific names of *Panax notoginseng* and *Coptis chinensis* were verified using http://www.theplantlist.org. According to the *Compendium of Materia Medica*, *Panax notoginseng* “treats all blood disorders, stops bleeding without leaving stasis, disperses blood stasis and relieves pain, reduces swelling and alleviates pain,” and has traditionally been employed to promote blood circulation, control bleeding, and alleviate pain and swelling, with widespread use in cardiovascular-related diseases (19, 20). *Panax notoginseng* possesses significant anti-inflammatory effects, which may exert therapeutic potential in pathological conditions such as myocardial ischemia and atherosclerosis (21–23). In addition, the abundant notoginsenosides in *Panax notoginseng* can activate autophagy in cardiomyocytes by enhancing the phosphorylation of AMPK and CaMKII, thereby providing cardioprotective effects and helping to prevent or mitigate MI and HF (24). The *Shennong Bencao Jing* records that *Coptis chinensis* is bitter, cold, and nontoxic, capable of clearing heart and abdominal heat, detoxifying, stopping diarrhea, and calm palpitations. Historically, it has been employed to clear heat, dispel dampness, detoxify, and calm the mind. The term “calm palpitations” traditionally refers to alleviating symptoms such as palpitations and irritability, which are related to modern cardiovascular dysfunction (25, 26). In China, *Coptis chinensis* is a frequent component in traditional herbal formulations used to treat cardiovascular-related conditions, exerting its effects through mechanisms such as its anti-inflammatory properties (27). In addition, *Coptis chinensis* is rich in coptisine, a compound with strong antioxidant activity, which can provide cardioprotective effects in isoproterenol-induced myocardial infarction rat models by suppressing the RhoA/Rho kinase signaling pathway (28).

Based on these pharmacological properties, the combination of *Panax notoginseng* and *Coptis chinensis* may exert synergistic effects by promoting blood circulation, removing blood stasis, clearing heat, and detoxifying. This could subsequently alleviate inflammation and cardiac stress, thereby offering potential cardioprotective benefits against MI. This study aims to provide further theoretical support for the role of THF in the treatment of MI and to demonstrate its potential as a therapeutic agent for this condition.

## 2 Materials and methods

### 2.1 Protocol for the preparation of THF extract and THF serum in rats

THF was prepared following a previously reported protocol (29). Briefly, *Panax notoginseng* and *Coptis chinensis* were purchased from the Guangdong Provincial Pharmaceutical Corporation (Traditional Chinese Medicine Decoction Pieces Factory). The herbal materials were authenticated by Guangdong Pharmaceutical University according to the identification standards described in the Pharmacopoeia of the People’s Republic of China (ISBN 2020, Volume I), and the results met all quality requirements. The herbs were ground into fine powder, and 400 g of each was extracted three times with 70% ethanol under reflux at 80°C for 2 h per extraction. The combined extracts were concentrated under reduced pressure to remove ethanol, dissolved in purified water, and further purified using D101 macroporous adsorption resin (Lanxiao, Xi’an, China). The purified solution was vacuum-dried at 60°C to obtain the final THF extract (30).

To prepare THF serum, twenty healthy adult male Sprague–Dawley (SD) rats were orally gavaged with THF extract at a dosage equivalent to 3 g/kg (calculated based on THF powder), administered twice daily over a period of three consecutive days. Following the final administration, blood samples were collected and the serum was separated by centrifugation. The resulting serum was heat-inactivated at 56°C for 30 minutes, sterilized through a 0.22 µm membrane filter, and subsequently stored at −80°C for later experimental use.

### 2.2 Construction and grouping of MI animal models

All animal procedures were performed in strict accordance with the WMA Statement on the Use of Animals in Biomedical Research and complied with the European Union Directive 2010/63/EU concerning animal welfare and experimental protocols. All animal procedures were performed in accordance with the guidelines of and approved by the Guangdong Pharmaceutical University’s Institutional Animal Care and Use Committee (Protocol No. gdpulacspf 2022256).

Male C57BL/6 mice, 6–8 weeks old and weighing 20–22 g, were obtained from ChangSheng Biotechnology (Liaoning, China). The animals were maintained under standard laboratory conditions, including a controlled temperature of 22 ± 1°C, relative humidity of 55 ± 5%, and a 12-hour light/dark cycle. After a five-day acclimatization period, MI was generated by irreversibly ligating the LAD artery under anesthesia with intraperitoneal pentobarbital sodium. The ligation site was positioned 2–3 mm distal to the origin of the left coronary artery, situated between the conus of the pulmonary artery and the left atrial appendage. Successful induction of myocardial infarction was confirmed by the presence of a visibly pale region in the affected area of the myocardium. After surgery, the animals were randomly assigned to six groups, including a control group (n = 25), an MI group (n = 25), three MI + THF groups (n = 25) treated with THF at doses of 60, 120, or 180 mg/kg/day by oral gavage, and an MI + fosinopril (FST) group (n = 25) administered 4.67 mg/kg/day fosinopril. The THF doses were selected according to previously published studies, which demonstrated their safety and feasibility in mice; low, medium, and high doses were therefore used in this study (31, 32). Both THF and FST were suspended in 0.5% sodium carboxymethylcellulose solution and administered once daily via gavage for a total duration of four weeks.

### 2.3 Echocardiography

Cardiac anatomy and function were examined using advanced high-resolution echocardiography (Vevo 2100, Fujifilm VisualSonics, Toronto, Canada) at the Animal Center of Guangdong Pharmaceutical University. Prior to imaging, mice were anesthetized with 3% isoflurane for induction, fixed in the supine position, and the chest area was depilated and coated with ultrasound coupling gel. During the imaging procedure, anesthesia was maintained with 1.5% isoflurane. Based on our previously established and published protocol, several key parameters of cardiac function were systematically evaluated, including ejection fraction (EF), fractional shortening (FS), and the left ventricular internal diameter measured at both end-diastole (LVIDd) and end-systole (LVIDs). For each mouse, measurements of all parameters were performed in triplicate to ensure accuracy and reproducibility, and the mean value of these repeated measurements was calculated. These averaged values were subsequently used for further quantitative analyses and statistical comparisons across experimental groups.

### 2.4 Enzyme-linked immunosorbent assay

Serum inflammatory and oxidative stress markers were quantified using commercial ELISA kits, including TNF-α (ml002095, Mlbio, Shanghai, China), IL-18 (ml002294, Mlbio), IL-1β (ml001554, Mlbio), and 8-OHdG (E-EL-0028, Elabscience).

### 2.5 LDH measurement

Lactate dehydrogenase (LDH) levels were quantified using a Lactate Dehydrogenase Assay Kit (BC0685, Solarbio, Beijing, China) in both serum and cellular samples according to the manufacturer’s specifications.

### 2.6 MDA measurement

Serum malondialdehyde (MDA) levels were determined using a Lipid Peroxidation Assay Kit (S0131S, Beyotime, Shanghai, China) according to the manufacturer’s instructions.

### 2.7 Immunohistochemistry assay

At 28 days after MI, mouse hearts were harvested, fixed in 4% paraformaldehyde, routinely paraffin-embedded, and serially sectioned at a thickness of 5 μm. Sections from the left ventricular myocardium were selected for subsequent histological analyses. Hematoxylin and eosin (H&E) staining was performed to evaluate myocardial morphology and structural alterations.

Cardiomyocyte cross-sectional area was assessed by fluorescent wheat germ agglutinin (WGA) staining (5 μg/mL, 25530, AAT Bioquest, USA). Individual WGA-labeled cardiomyocytes were manually outlined to determine cross-sectional area. For each sample, three randomly selected fields were analyzed, and the average cardiomyocyte cross-sectional area per field was calculated using ImageJ software.

Immunohistochemical staining was performed to examine the expression and spatial distribution of inflammation-related proteins. Paraffin-embedded sections were deparaffinized and rehydrated, followed by antigen retrieval and blocking, and then incubated with primary antibodies at 4°C overnight. Primary antibodies included anti-cGAS (H651468009, Huabio, 1:200), anti-cleaved caspase-1 (WL02996a, Wanleibio, 1:200), and anti-NLRP3 (ab283819, Abcam, 1:200). Sections were subsequently incubated with HRP-conjugated secondary antibodies (ab205718, Abcam, 1:500), visualized using DAB substrate (DA1010, Solarbio), and counterstained with hematoxylin. All stained sections were imaged using an Olympus DX51 microscope.

### 2.8 Cell culture and treatments

H9c2 cardiomyoblasts (Procell, China) were cultured in DMEM supplemented with 10% FBS and 1% penicillin–streptomycin (Gibco, USA) under standard conditions. Primary neonatal mouse cardiomyocytes (NMCMs) were isolated from 1–3-day-old SD rats (Zhuhai Best-Test Bio-Tech, China) using enzymatic digestion and maintained in DMEM containing 5% FBS.

For THF serum treatment, cardiomyocytes were exposed to DMEM containing 12% THF serum or fosinopril. Oxidative stress was induced in NMCMs using hydrogen peroxide (up to 400 μM). STING pathway activation was achieved by treating cells with cGAMP (2 μg/mL; HY-100564-A, Med Chem Express, USA). The cGAS inhibitor RU.521 was applied at 10 μM following preparation of a DMSO stock solution, with DMSO-treated cells serving as controls.

After the indicated treatments, cells were collected for RNA extraction, viability assays, cell death analysis, protein isolation, or fluorescence staining.

### 2.9 CCK-8 assay

NMCMs were seeded in 96-well plates and cultured for 24 h. Cells were then treated with different concentrations of THF-containing serum or H_2_O_2_. Cell viability was evaluated using the CCK-8 assay, with absorbance measured at 450 nm after incubation.

### 2.10 Western blotting

Sixty micrograms of protein extracted from cells or tissues were separated by SDS-PAGE and transferred onto nitrocellulose membranes (HATF00010, Merck, Germany). Following blocking, the membranes were incubated overnight at 4 °C with the primary antibodies listed in **Table 1**, washed with PBST, and then incubated with a fluorescent anti-rabbit IgG secondary antibody (1:8000; 926-32211, LI-COR Biosciences, USA). Protein bands were visualized and quantified using an Odyssey imaging system.

**Table 1:** Primary antibodies for western blotting.

Anti-body	Company	Catalog number	Dilution ratio
cGAS	CellSignaling Technology	31659s	1:1000
cGAS	HUABIO	H651468009	1:1000
P-TBK1	CellSignaling Technology	5483s	1:1000
TBK1	Abcam	AB40676	1:1000
P-IRF3	CellSignaling Technology	29047s	1:1000
IRF3	ABclonal	A2172	1:1000
GSDMDNT/GSDMD	Abcam ABclonal	AB283819 WH240405	1:1000 1:1000
NLRP3	Abcam	Ab283819	1:1000
Cleave-caspase1	Wanleibio	WL02996a	1:1000
Nrf2	Proteintech	16393-1-AP	1:1000
Keap1	Proteintech	60027-1-AP	1:1000
Nox4	GeneTex	GTX121929	1:1000
GAPDH	Proteintech	60004–1-lg	1:2000

### 2.11 Total RNA isolation and quantitative real-time PCR

Total RNA was extracted from cardiac tissues and myocardial cells using TRIzol reagent (15596018, Invitrogen, USA) and RNA concentration was measured using a microplate reader (33). Complementary DNA (cDNA) was synthesized using the ReverTra Ace qPCR RT kit (Toyobo, Japan; FSQ-101). For qPCR, cDNA was mixed with SYBR Green and primers, and amplification and signal detection were performed on an ABI 7500 Fast Real-Time PCR System (Applied Biosystems, CA, USA). Primer sequences are listed in **Supplementary Table S1**. Relative mRNA expression levels were calculated using the 2^−ΔΔCt method, with GAPDH serving as the internal control.

### 2.12 Evaluation of mitochondrial membrane potential

Mitochondrial membrane potential (Δψm) was assessed using a JC-1 staining kit (C2006, Beyotime, Shanghai, China) following the manufacturer’s instructions. Δψm changes were visualized using an Olympus fluorescence microscope.

### 2.13 Immunofluorescence staining

H9c2 cells were subjected to TUNEL staining using the TUNEL Fluorescence FITC Kit (Roche, Indianapolis, IN, USA) to assess apoptosis. Caspase-1 expression was detected by immunofluorescence using a primary antibody (Affinity, 1:500) and an Alexa Fluor 594-conjugated secondary antibody (Invitrogen, 1:200). Nuclei were counterstained with DAPI, and fluorescence images were acquired using an Olympus microscope.

### 2.14 Screening of compounds in THF and prediction of potential targets

To identify the bioactive ingredients of THF, we performed a screening using the TCMSP database (34). Compounds were screened for inclusion if they exhibited an oral bioavailability of at least 30% and a drug-likeness value of 0.18 or higher. The structural information of the active components was then retrieved from the PubChem database and converted into SMILES format (35). The SwissTargetPrediction database was subsequently populated with the SMILES files. From this platform, potential biological targets exhibiting a probability score greater than zero were chosen (36).

For the subsequent identification of disease-related targets, the term “myocardial infarction” was employed to query the GeneCards database, selecting targets with a relevance score above 5.0 (37) and from the OMIM database (38). The extracted data were merged to obtain a comprehensive list of disease-associated targets. The intersection of drug targets and disease targets was identified using the Venny tool (39), and a Venn diagram was constructed. The overlapping targets were considered potential targets.

A “drug-active compound-potential target” network was constructed using Cytoscape 3.9.1 (40). The network included THF, its active compounds, and potential targets. Node size and color were adjusted based on degree values. Subsequently, the 126 potential targets were imported into the STRING database to construct a protein–protein interaction (PPI) network. The interaction data were then analyzed using Cytoscape 3.9.1, and core targets were identified based on degree values (41).

The identified potential targets were analyzed using the DAVID database to perform comprehensive enrichment analyses for Gene Ontology (GO) terms and KEGG (Kyoto Encyclopedia of Genes and Genomes) pathways (42). The GO enrichment analysis specifically focused on biological processes (BP), with statistical significance defined as *P* < 0.05. Subsequently, the top five significant BP terms and ten KEGG pathways of interest were visualized using R software.

### 2.15 Microarray data expression analysis

We sourced all microarray gene expression data utilized in this research from the Gene Expression Omnibus (GEO) database (43). The dataset GSE66360, which is annotated based on the GPL570 platform, includes samples from patients with acute myocardial infarction (n = 49) and healthy controls (n = 50). The identification of differentially expressed genes (DEGs) was conducted by applying the “limma” package in R environment (version 4.4.1), with the selection criteria set as |logFC| > 0.5 and P-value < 0.05.

### 2.16 Molecular docking study

Molecular docking analyses were conducted for the active compounds of THF. The three-dimensional structures of cGAS (PDB ID: 8SHZ) and Keap1 (PDB ID: 4IFJ) were obtained from the PDB database (44). The molecular structures of the active compounds were retrieved from the TCMSP database and appropriately optimized, including the addition of hydrogen atoms and adjustment of charge distribution. The receptor protein structures were standardized, including the removal of water molecules and addition of polar hydrogens. The prepared ligands and receptors were then subjected to molecular docking using AutoDock Vina to predict binding conformations and affinities. The resulting docking poses were visualized in PyMOL (version 2.6.0) for the analysis of potential key interactions.

### 2.17 Statistical analyses

All data are expressed as mean ± standard error of the mean (SEM). Group differences were evaluated using one-way ANOVA followed by suitable *post hoc* tests. A P value less than 0.05 was considered statistically significant. Statistical analyses and figure generation were conducted with GraphPad Prism 10.0.

## 3 Results

### 3.1 THF improved cardiac function of mice post-MI

**Figure 1:**
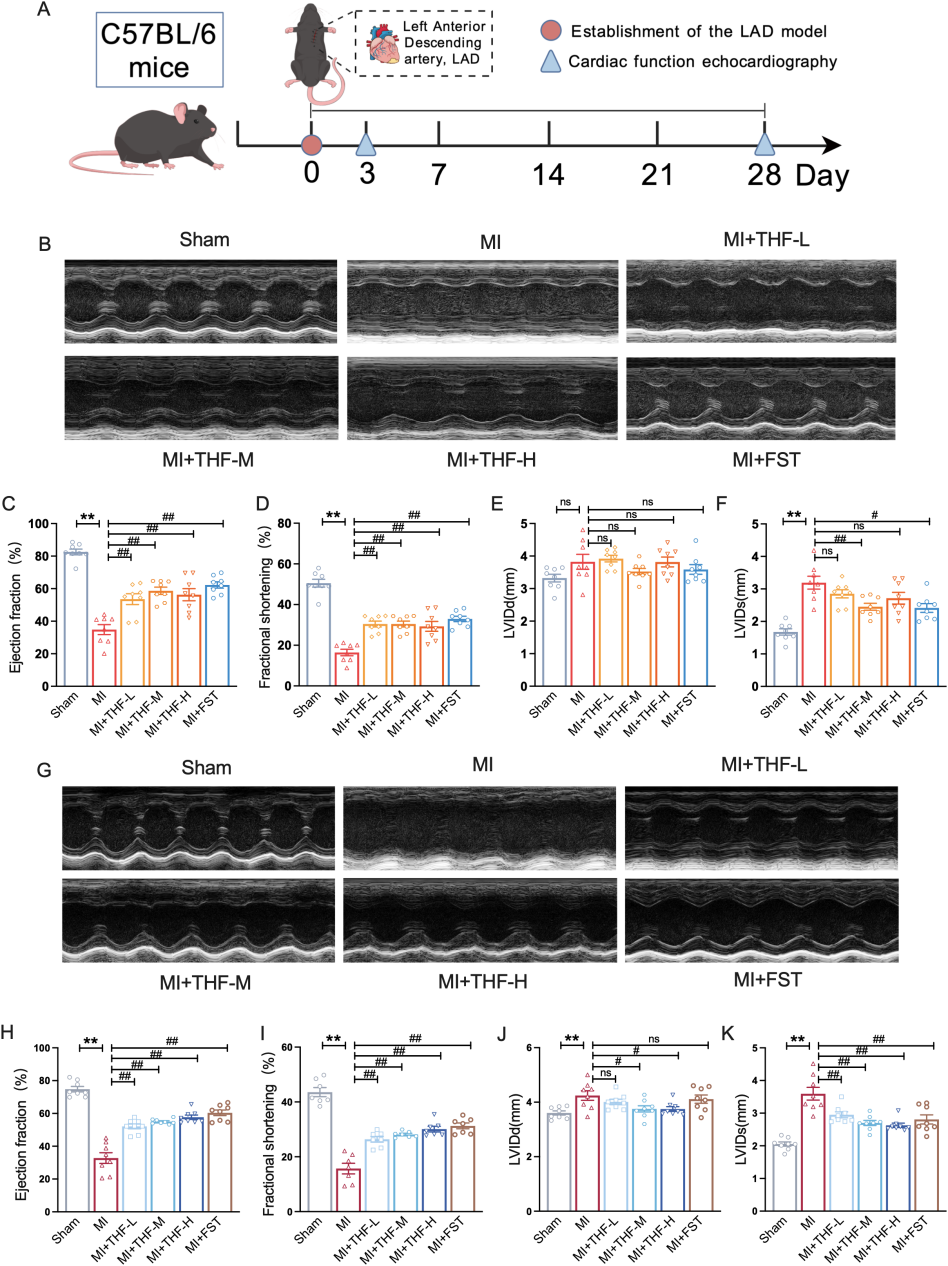
THF improved cardiac function of mice post-MI. **(A)** After MI modeling, mice were treated with THF or FST for 28 days, and cardiac function was assessed on days 3 and 28 post-MI. **(B)** Representative echocardiographic images of mice on day 3 post-MI. **(C–F)** Quantitative data of EF, FS, LVIDd, and LVIDs of MI mice on day 3 post-MI (n = 8). **(G)** Representative echocardiographic images of mice on day 28 post-MI. **(H–K)** Quantitative data of EF, FS, LVIDd, and LVIDs in MI mice on day 28 post-MI (n = 8). ^*^P < 0.05, ^**^P < 0.01 vs. Sham group; ^#^*P* < 0.05, ^##^*P* < 0.01 vs. MI group.

To investigate the therapeutic effects of THF in mice with MI, we assessed cardiac function via echocardiography at two critical time points (**Figure 1A**): day 3 (acute phase, **Figures 1B–F**) and day 28 (chronic remodeling phase, **Figures 1G–K**) post-MI (45, 46). FST was chosen as the positive drug (47, 48). The results demonstrated that both THF and FST treatments significantly preserved cardiac function in post-MI mice, as evidenced by higher EF and FS compared to the untreated MI group at day 3 (**Figures 1C, D**) and day 28 (**Figures 1H, I**). In addition, both THF and FST attenuated adverse ventricular remodeling. On day 3 post-MI, treatment significantly reduced LVIDs (**Figures 1E, F**). By day 28, however, both LVIDd and LVIDs were markedly decreased in the THF- and FST-treated groups (**Figures 1J, K**). These findings suggest that THF exerts cardioprotective effects by improving systolic function and limiting pathological dilation, two key determinants of post-MI outcomes.

### 3.2 THF attenuated post-MI cardiac hypertrophy and inflammatory response

**Figure 2:**
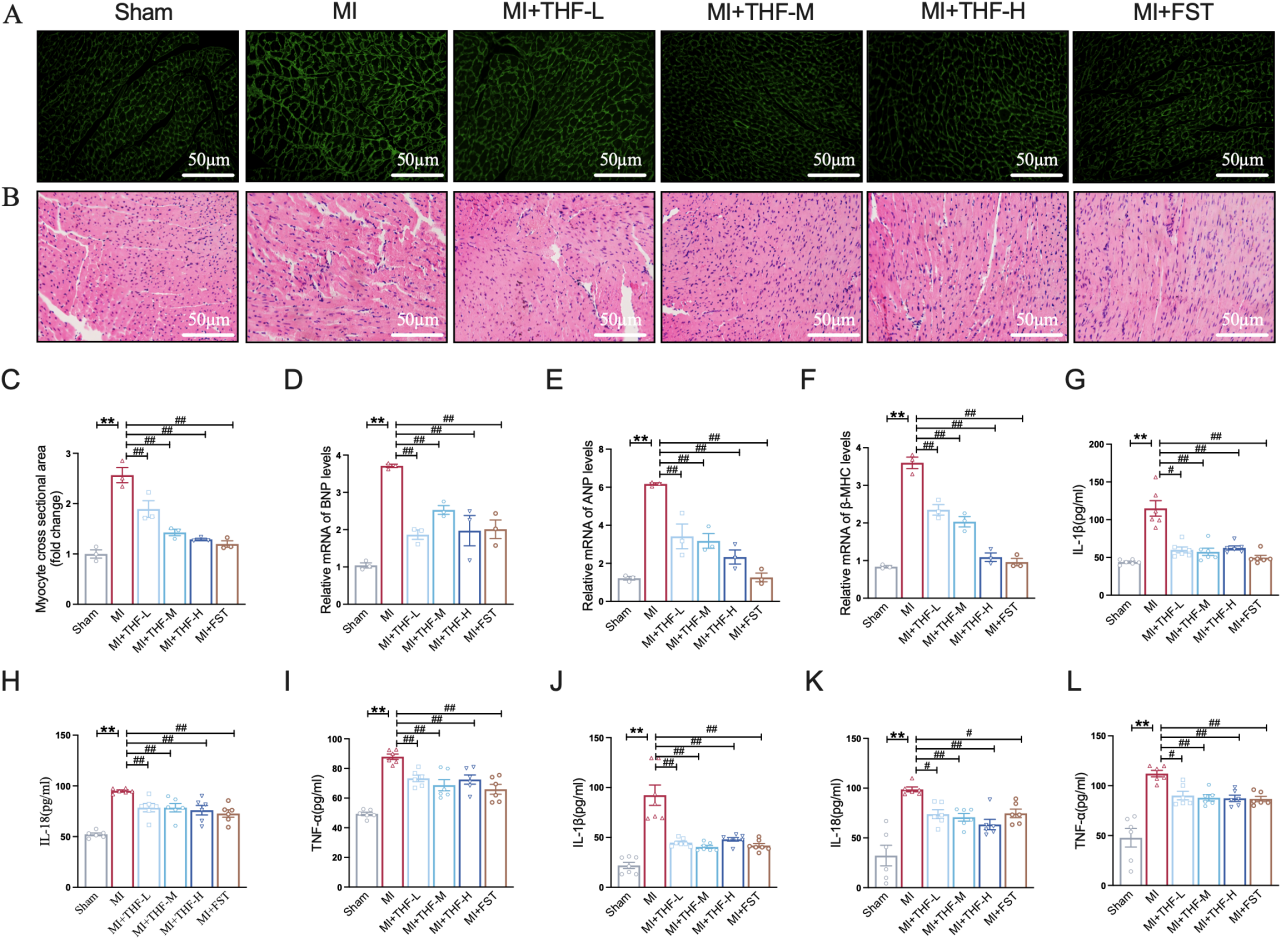
THF attenuated post-MI cardiac hypertrophy and inflammatory response. **(A)** Representative WGA staining images (n = 3). **(B)** Representative H&E staining images (n = 3). **(C)** Quantification of cardiomyocytes cross-sectional area using WGA staining (n = 3). **(D–F)** Cardiac tissue mRNA expression of ANP, BNP, and β-MHC was quantified (n = 3). **(G–I)** ELISA analysis of serum IL-1β, IL-18, and TNF-α levels in MI mice day 3 post-MI (n = 6). **(J–L)** ELISA analysis of serum IL-1β, IL-18, and TNF-α levels in MI mice day 28 post-MI (n = 6). ^*^P < 0.05, ^**^P < 0.01 vs. Sham group; ^#^*P* < 0.05, ^##^*P* < 0.01 vs. MI group.

Following MI, compensatory myocardial hypertrophy occurs in response to injury and functional loss, a maladaptive process that often progresses to HF (49). To evaluate the therapeutic effects of THF on pathological remodeling, we analyzed cardiac hypertrophy and inflammation at day 28 post-MI. A marked enlargement in the cross-sectional area of cardiomyocytes was observed in the MI group relative to sham controls, a finding from WGA staining that suggests pathological hypertrophy. However, THF and FST treatments markedly reduced this hypertrophic response (**Figure 2A**). Consistent with these findings, quantitative RT-PCR demonstrated that THF dose-dependently downregulated the expression of hypertrophy-associated genes (ANP, BNP, and β-MHC) (**Figures 2D–F**), further supporting its anti-hypertrophic effects. In addition, H&E staining revealed pronounced structural disorganization and extensive infiltration of inflammatory cells in MI hearts, which was markedly alleviated by THF treatment (**Figure 2B**). To quantify systemic inflammation, we measured mouse serum levels of TNF-α, IL-1β, IL-18 via ELISA. At both day 3 (**Figures 2G–I**) and 28 (**Figures 2J–L**) post-MI, THF robustly suppressed cytokine elevation, suggesting sustained anti-inflammatory activity. These results suggested that THF ameliorates inflammatory cell infiltration and myocardial hypertrophy induced by MI.

### 3.3 Integrated network pharmacology and transcriptomic analysis of THF in MI

**Figure 3:**
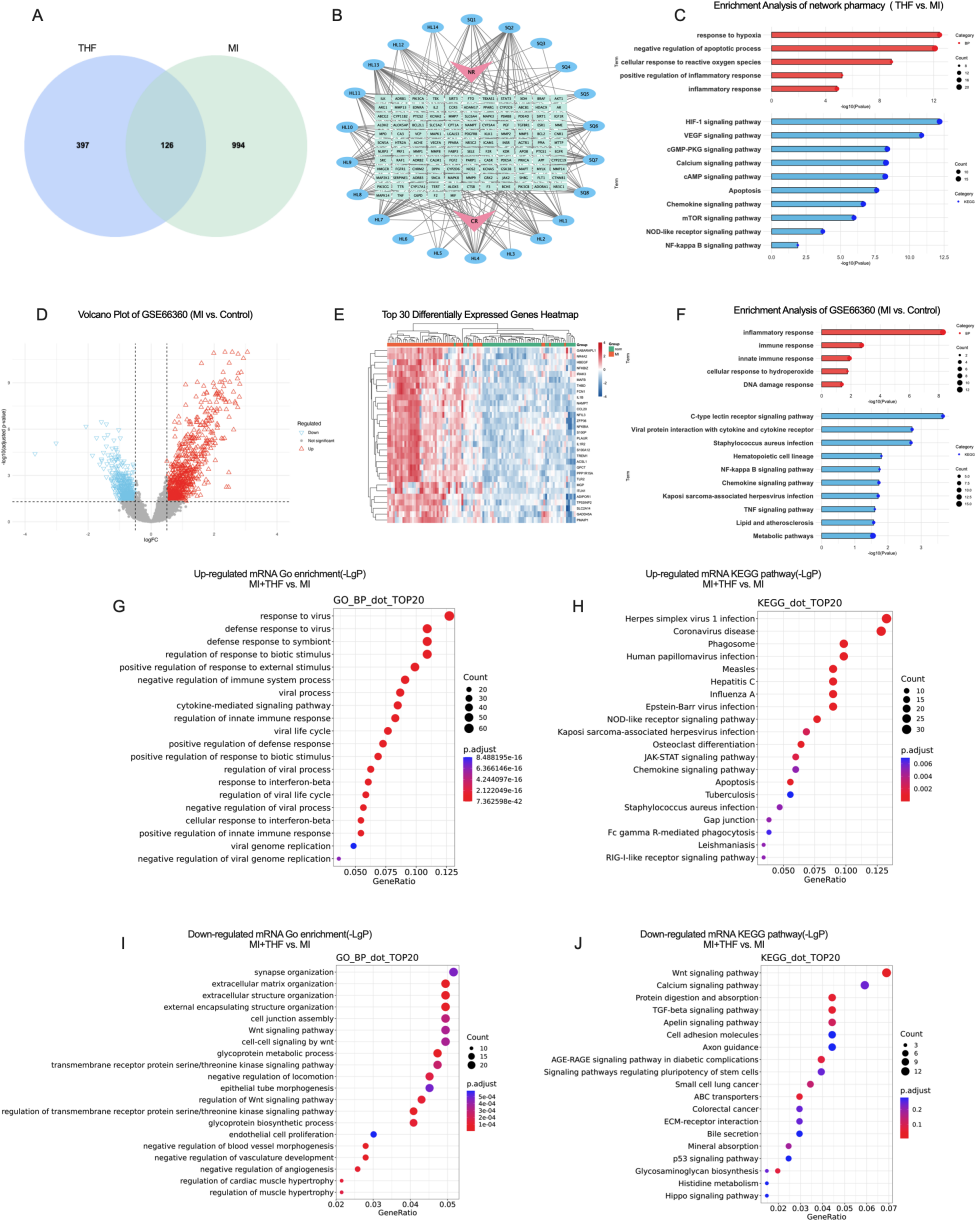
Integrated network pharmacology and transcriptomic analysis of THF in MI. **(A)** Venn diagram showing 126 IGEs between THF-associated targets and MI-related genes. **(B)** Network diagram depicting the “herb–compound–target” interactions, highlighting the multi-component and multi-target pharmacological characteristics of THF. **(C)** GO and KEGG pathway enrichment analysis of the 126 intersecting genes, revealing BP and signaling pathways potentially modulated by THF in MI. **(D, E)** Volcano plot **(D)** and heatmap **(E)** of the top 30 significant DEGs between MI and control groups based on the GSE66360 dataset. **(F)** GO and KEGG pathway enrichment analysis of the 49 overlapping genes, reflecting shared pathological mechanisms and potential therapeutic targets of THF in MI. **(G, H)** GO **(G)** and KEGG **(H)** Enrichment analyses of mRNAs upregulated in the MI+THF group relative to the MI group, indicating potential regulatory effects of THF at the transcriptomic level. **(I, J)** GO **(I)** and KEGG **(J)** Enrichment analyses of mRNAs downregulated in the MI+THF group relative to the MI group, indicating potential regulatory effects of THF at the transcriptomic level.

To explore the potential mechanisms underlying the cardioprotective effects of THF in MI, an integrative analysis combining network pharmacology based on human gene targets with transcriptomic profiling was performed. Twenty-one putative active compounds in THF were identified using the TCMSP database, and their potential targets were predicted, yielding a total of 523 candidate genes, including 8 compounds derived from *Panax notoginseng* and 14 compounds derived from *Coptis chinensis*. Intersection of these predicted targets with 1,120 MI-related genes resulted in 126 putative therapeutic targets (**Figure 3A**). A herb–compound–target network was constructed (**Figure 3B**), indicating the multi-component and multi-target regulatory characteristics of THF. Core PPI nodes were mainly enriched in immune- and inflammation-related molecules, including AKT1, TNF, STAT3, and PTGS2, suggesting a potential role of THF in modulating inflammatory responses (**Supplementary Figure S1**).

GO and KEGG enrichment analyses showed that these targets were mainly involved in immune and inflammatory regulation and oxidative stress–related biological processes. Enriched pathways included pyroptosis, the mTOR signaling pathway, the NOD-like receptor signaling pathway, and the NF-κB signaling pathway (**Figure 3C**), suggesting prominent functional enrichment of THF-related targets in immune- and stress-associated pathways.

Analysis of the publicly available transcriptomic dataset GSE66360 identified 1,245 differentially expressed genes in MI patients, which were visualized by volcano plots and heatmaps (**Figures 3D–E**). When these differentially expressed genes were intersected with THF-related targets, the overlapping genes were predominantly enriched in innate immune responses, DNA damage response, and inflammation-related biological processes (**Figure 3F**), indicating that immune and stress responses highlighted by network pharmacology are markedly activated in MI.

Transcriptomic profiling performed at 28 days after MI induction, corresponding to the ventricular remodeling phase in a mouse MI model, revealed that THF markedly upregulated biological processes related to immune and inflammatory responses, including responses to virus, regulation of innate immune responses, and cytokine-mediated signaling pathways. The corresponding KEGG pathways were mainly associated with virus-related diseases, the NOD-like receptor signaling pathway, programmed cell death, and the JAK–STAT signaling pathway (**Figures 3G, H**). In contrast, THF significantly downregulated biological processes related to cardiac hypertrophy, extracellular matrix production, and tissue remodeling, with enriched pathways including Wnt, TGF-β, and calcium signaling pathways (**Figures 3I, J**), suggesting an attenuation of pathological ventricular remodeling.

Notably, in the absence of exogenous viral infection, the virus-like innate immune response signatures observed in MI transcriptomes are more likely to reflect damage-associated molecular pattern–mediated immune activation. Together with the significant enrichment of differentially expressed genes in DNA damage response, NOD-like receptor signaling, and programmed cell death–related processes, these findings indicate an innate immune activation state in MI myocardium driven by endogenous nucleic acid damage signals and functionally coupled to inflammasome formation and pyroptosis. Substantial evidence indicates that myocardial ischemic injury leads to the release of nuclear and mitochondrial DNA into the cytoplasm, thereby activating nucleic acid-sensing innate immune pathways (50, 51). Among these pathways, the cGAS–STING axis plays a central regulatory role in linking DNA release induced by cellular injury with oxidative stress, mitochondrial dysfunction, NLRP3 inflammasome activation, mTOR signaling regulation, and type I interferon responses (11, 52, 53). Based on transcriptomic enrichment patterns and its well-established role in the regulation of inflammatory injury, the cGAS–STING pathway was considered a putative upstream signaling axis with potential biological relevance and was therefore selected for further mechanistic investigation.

### 3.4 THF effectively mitigated the oxidative stress and over-activated cGAS-STING signaling pathway in the heart of post-MI mice

**Figure 4:**
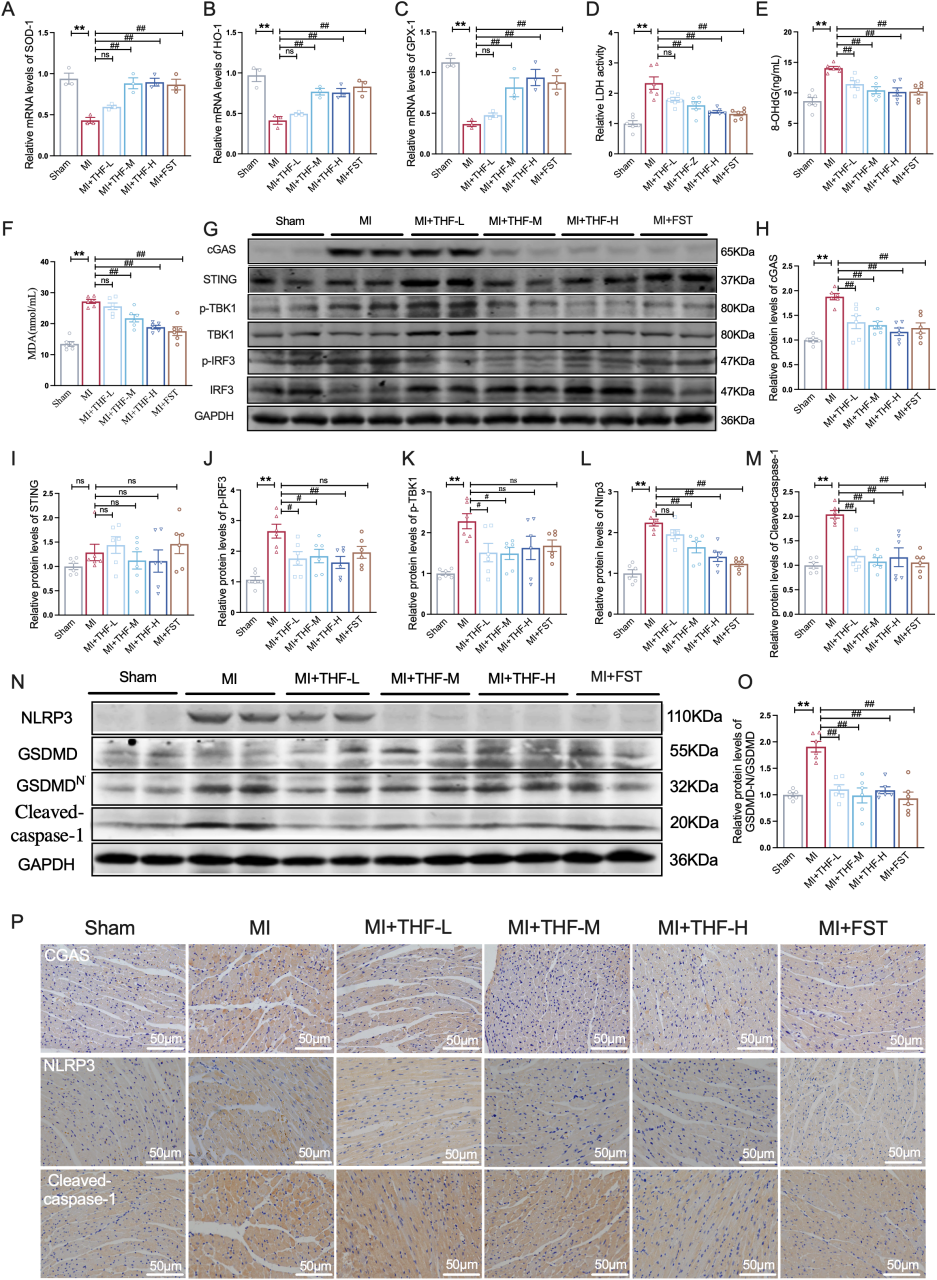
THF effectively mitigated the oxidative stress and over-activated cGAS-STING signaling pathway in the heart of post-MI mice. **(A–C)** qRT-PCR analysis of SOD-1, HO-1, and GPX-1 mRNA expression levels in cardiac tissue (n = 3) **(D)** Measurement of LDH release levels in mouse serum (n = 3). **(E)** Measurement of 8-OHdG levels in mouse serum. **(F)** Evaluation of MDA levels in mouse serum (n = 3). **(G–K)** Western blotting was employed to determine the protein expression of cGAS, STING, p-IRF3/IRF3, and p-TBK1/TBK1 within cardiac tissue. (n = 6). **(K–O)** Western blotting was employed to determine the protein expression of NLRP3, Cleaved-caspase-1, and GSDMD-N/GSDMD within cardiac tissue. (n = 6). **(P)** Immunohistochemical staining analysis of cGAS, NLRP3, and Cleaved-caspase-1 expression in cardiac tissue (n = 3). ^*^P < 0.05, ^**^P < 0.01 vs. Sham group; ^#^*P* < 0.05, ^##^*P* < 0.01 vs. MI group.

The cGAS–STING signaling pathway is a critical regulator of innate immune–mediated inflammatory responses. By sensing cytosolic DNA and initiating downstream inflammatory cascades, this pathway plays a pivotal role in the inflammation and cellular injury that follow MI. Notably, MI is accompanied by pronounced oxidative stress, which not only induces DNA damage but also promotes the release of mitochondrial DNA into the cytoplasm, thereby serving as a potent trigger for cGAS–STING activation. To thoroughly assess the impact on oxidative stress and injury, we validated the mRNA levels of crucial antioxidant factors using qRT-PCR. The results showed that the levels of antioxidant factors, including SOD-1, HO-1, and GPX-1, were significantly downregulated in MI mice. However, these detrimental changes were effectively reversed following THF treatment (**Figures 4A–C**). Furthermore, to gain a comprehensive understanding of myocardial injury and redox imbalance in mice, we assessed several serum biomarkers associated with oxidative stress, consisting of LDH, a key enzyme released following cell membrane disruption, serves as a sensitive indicator of cell pyroptosis; MDA, a major end-product of lipid peroxidation, directly reflects oxidative degradation of membrane lipids, as well as 8-OHdG, a reliable marker of oxidative DNA damage. As results shown in **Figures 4D–F**, the levels of LDH, 8-OHdG and MDA were notably increased in serums of MI mice, which were all alleviated by THF treatment. Furthermore, the classic pathway Nox4/Keap1/Nrf2, which plays a crucial role in regulating cellular redox balance and antioxidant responses, were also detected. Our results demonstrated increased levels of Nox4 and Keap1 with a decreased Nrf2 in the MI mice hearts, indicating a compromised endogenous antioxidant defense system. While they were effectively reversed after THF administration (**Supplementary Figure S2**). Subsequently, we assessed the expression of key proteins in the cGAS–STING pathway in cardiac tissues from MI mice. Compared with the sham group, MI mice exhibited markedly increased cGAS, p-TBK1, p-IRF3 protein expression, with the unchanged protein level of TBK1, IRF3, and STING (**Figures 4G–K**), confirming the central involvement of this pathway in ischemic injury–induced myocardial inflammation. Importantly, THF treatment significantly attenuated the aberrant upregulation of these proteins (**Figures 4G–K**). Numerous studies have demonstrated that MI-induced cGAS-STING activation promotes the NLRP3 inflammasome, leading to pyroptosis. This intricate process involves caspase-1 activation, which cleaves pro-IL-1β to mature IL-1β and induces GSDMD-N formation, thereby promoting inflammation and cell death (49). Our results demonstrated that the expression levels of NLRP3 inflammasome-related proteins were significantly elevated in the cardiac tissues of MI mice, while effectively reversed after THF administration (**Figures 4L–O**). Concurrently, immunohistochemical staining further corroborated these findings, showing that THF significantly suppressed the expression of cGAS, NLRP3, and Cleaved-caspase-1 proteins in MI-post cardiac tissues (**Figure 4P**). These comprehensive results strongly suggested that THF effectively mitigated MI-induced oxidative stress, which subsequently attenuated the activation of the cGAS-STING pathway and downstream NLRP3 inflammasome, thereby mediating cardiac immunoinflammation and improving cardiac function.

### 3.5 THF exerted antioxidant and anti-inflammatory effects in H_2_O_2_-treated cardiomyocytes

To elucidate the effects of THF *in vitro*, we established a hypoxia-mimicking injury model in primary NMCMs using H_2_O_2_, based on previous studies (54). In addition, sterile rat serum containing THF (THF-serum) was prepared to evaluate the regulatory effects of THF on NMCMs (55). To identify the optimal H_2_O_2_ concentration, primary NMCMs received escalating doses of the compound. The results showed a dose-dependent decline in cell viability, with 400 μmol/L identified as the optimal concentration that induced significant cellular injury while retaining acceptable cell viability (**Supplementary Figure S3**). Based on this model, the effects of different concentrations of THF-serum on injured NMCMs were further evaluated. Among the tested concentrations, 12% THF-serum produced the most pronounced improvement in cell viability and was therefore selected as the treatment concentration for subsequent experiments (**Supplementary Figure S4**).

**Figure 5:**
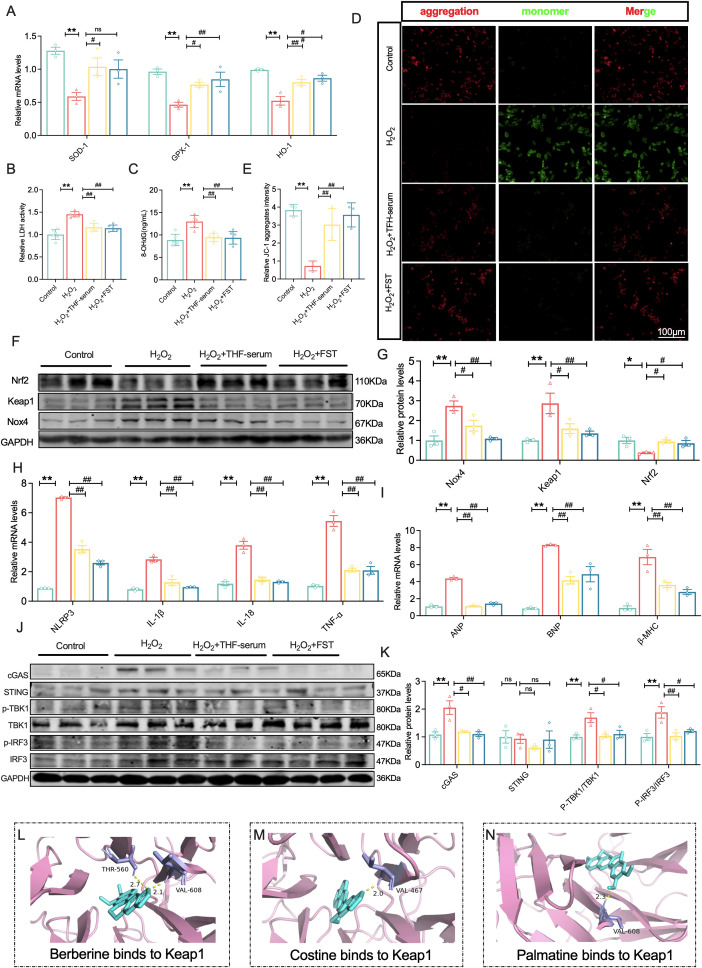
THF exerted antioxidant and anti-inflammatory effects in H_2_O_2_-treated cardiomyocytes. **(A)** mRNA expression levels of SOD-1, HO-1, and GPX-1 in NMCMs (n = 3). **(B)** LDH release in NMCMs (n = 6). **(C)** ELISA analysis of 8-OHdG expression in NMCMs (n = 6). **(D, E)** Representative micrographs of JC-1 staining in H9c2 rat cardiomyoblasts (n=3 per group). **(F–G)** Western blotting was employed to determine the protein expression of Nox4, Keap1,and Nrf2 within NMCMs. (n = 3). **(H)** mRNA expression levels of NLRP3, IL-1β, IL-18, and TNF-α in NMCMs (n = 3).**(I)** mRNA expression levels of ANP, BNP, and β-MHC in NMCMs (n = 3). **(J-K)** Western blotting was employed to determine the protein expression of cGAS, STING, p-IRF3/IRF3, and p-TBK1/IRF3 within NMCMs. (n = 3) **(L)** Berberine binding to Keap1. **(M)** Coptisine binding to Keap1. **(N)** Palmatine binding to Keap1. ^*^P < 0.05, ^**^P < 0.01 vs. the Control group; ^#^*P* < 0.05, ^##^*P* < 0.01 vs. the H_2_O_2_ group.

Functional assays revealed that THF-serum significantly upregulated the mRNA expression of antioxidant enzymes SOD-1, HO-1, and GPX-1, while reducing the levels of oxidative stress markers LDH and 8-OHdG (**Figures 5A–C**), indicating pronounced antioxidant effects. JC-1 immunofluorescence staining showed that H_2_O_2_ induced mitochondrial depolarization in H9c2 cells, evidenced by decreased red JC-1 aggregates and increased green monomers, whereas THF-serum treatment markedly attenuated this injury, suggesting a protective role in maintaining mitochondrial membrane potential (MMP) (**Figures 5D, E**). At the molecular level, H_2_O_2_ treatment significantly upregulated the pro-oxidant genes Nox4 and Keap1 while downregulating the antioxidant transcription factor Nrf2, suggesting impairment of endogenous antioxidant defense. THF-serum effectively reversed these abnormalities, restoring them toward control levels (**Figures 5F, G**).

In addition, H_2_O_2_ markedly increased the expression of inflammatory mediators NLRP3, TNF-α, IL-1β, and IL-18, whereas THF-serum significantly suppressed their levels (**Figure 5H**). Regarding cardiac hypertrophy, H_2_O_2_ treatment significantly upregulated the mRNA levels of ANP, BNP, and β-MHC, while THF-serum effectively reversed their aberrant expression, indicating its anti-hypertrophic effects (**Figure 5I**).

Mechanistically, H_2_O_2_ treatment markedly increased the levels of phosphorylated cGAS, p-TBK1, and p-IRF3 in NMCMs, while THF-serum effectively suppressed the activation of this pathway without altering total protein levels (**Figures 5J, K**), further supporting its anti-inflammatory role. Moreover, molecular docking results showed that Berberine (–9.3 kcal/mol) primarily formed hydrogen bonds with Keap1 residues THR-560 and VAL-608, Coptisine (–9.8 kcal/mol) achieved stable binding via interaction with VAL-467, and Palmatine (–8.1 kcal/mol) interacted with VAL-608 through hydrogen bonding. Collectively, these findings suggest that these alkaloids may inhibit Keap1 activity by targeting its critical amino acid residues (**Figures 5L–N**).

In conclusion, this study demonstrates that THF-serum attenuates H_2_O_2_-induced oxidative stress, restores mitochondrial function, suppresses cGAS–STING pathway activation and inflammasome hyperactivation, and may further enhance Nrf2-mediated antioxidant responses through direct interactions of its major alkaloid components with Keap1. Altogether, these effects contribute to its comprehensive cardioprotective actions against oxidative stress, inflammation, and myocardial hypertrophy.

### 3.6 THF mitigates cardiomyocyte inflammation and pyroptosis by inhibiting the cGAS-STING signaling pathway

**Figure 6:**
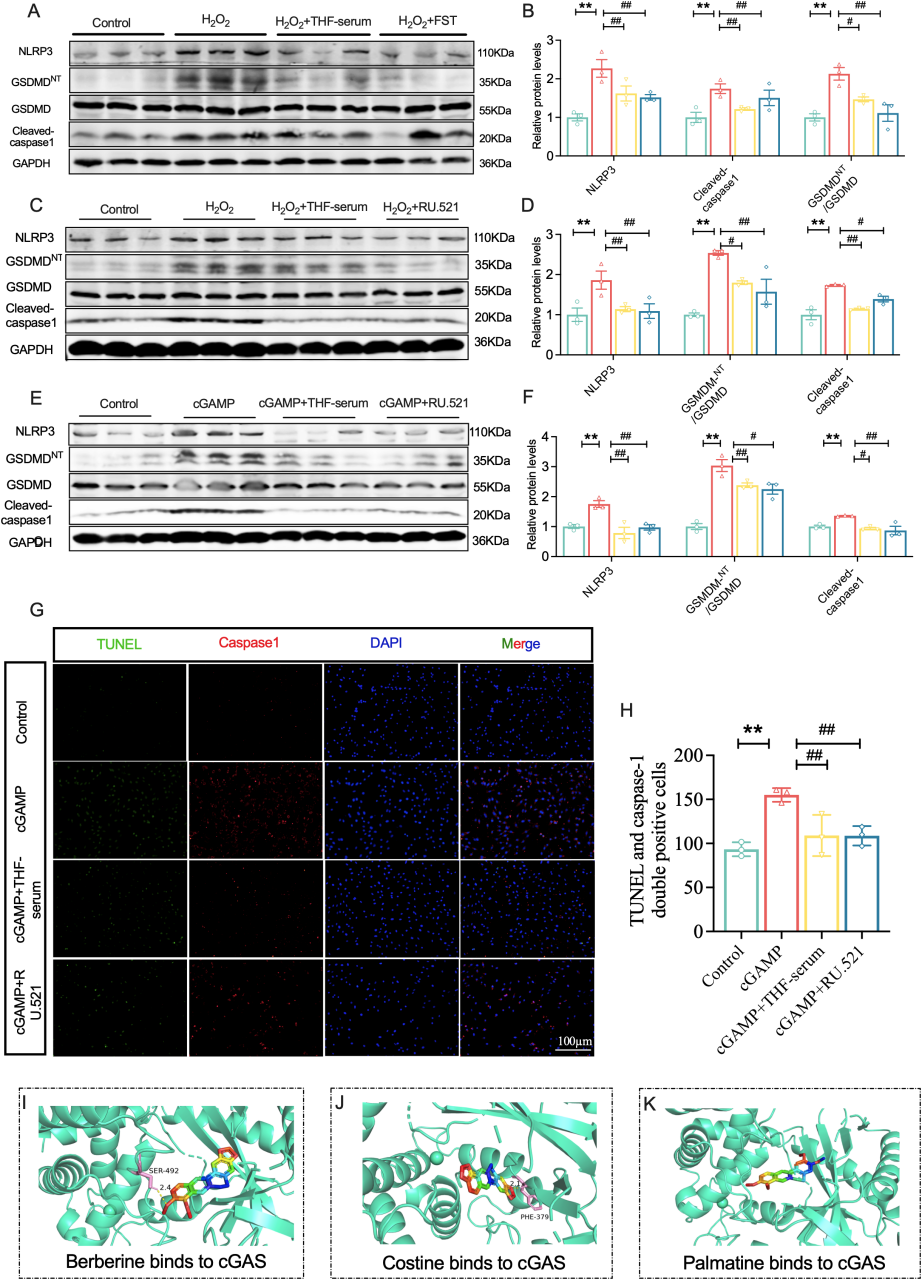
THF mitigates cardiomyocyte inflammation and pyroptosis by inhibited the cGAS-STING signaling pathway. **(A, B)** Western blotting was employed to determine the protein expression of NLRP3, Cleaved-caspase-1, and GSDMD-N/GSDMD in NMCMs following H_2_O_2_ stimulation (n = 3). **(C, D)** Western blotting was employed to determine the protein expression of NLRP3, Cleaved-caspase-1, and GSDMD-N/GSDMD in NMCMs following H_2_O_2_ stimulation and treatment with the cGAS inhibitor RU.521 (n = 3). ^*^P < 0.05, ^**^P < 0.01 vs. the Control group; ^#^*P* < 0.05, ^##^*P* < 0.01 vs. the H_2_O_2_ group. **(E, F)** Western blotting was employed to determine the protein expression of NLRP3, cleaved-caspase-1, and GSDMD-N/GSDMD in NMCMs following cGAMP stimulation and RU.521 treatment (n = 3). **(G, H)** Evaluation of pyroptosis activation in H9c2 cells using caspase-1 and TUNEL double staining (n = 3). ^*^P < 0.05, ^**^P < 0.01 vs. the Control group; ^#^*P* < 0.05, ^##^*P* < 0.01 vs. the cGAMP group. **(I)** Berberine binding to cGAS. **(J)** Coptisine binding to cGAS. **(K)** Palmatine binding to cGAS.

To clarify whether THF exerts its effects by inhibiting the cGAS–STING axis and downstream NLRP3 inflammasome activation, we further examined key NLRP3 inflammasome-related proteins, including NLRP3, GSDMD-N/GSDMD, and Cleaved-caspase-1. Exposure to H_2_O_2_ significantly elevated the levels of key pyroptosis-related proteins relative to controls, however, these changes were effectively reversed by treatment with THF-serum (**Figures 6A, B**). To further verify the involvement of pyroptosis, TUNEL and caspase-1 double immunofluorescence staining was performed. H_2_O_2_ treatment led to enhanced caspase-1 activity and an increased number of TUNEL-positive H9c2 cells, indicating elevated pyroptosis, whereas THF-serum treatment markedly reduced caspase-1 activation and TUNEL positivity (**Supplementary Figure S5**).

Selective cGAS inhibitor RU.521 was used as a positive control. RU.521 competitively binds to and inhibits cGAS activity (56). Both RU.521 and THF-serum pretreatment significantly suppressed the expression of NLRP3, GSDMD-N, and Cleaved-caspase-1, in primary NMCMs (**Figures 6C, D**), suggesting that THF exerts anti-inflammatory effects similar to those of cGAS inhibitors. To further validate this pathway, STING agonist cGAMP was employed to activate the cGAS–STING axis in NMCMs. cGAMP, an endogenous cyclic dinucleotide synthesized by cGAS upon cytosolic DNA stimulation, serves as a second messenger that directly binds and activates STING, thereby triggering downstream inflammatory signaling. cGAMP treatment significantly upregulated NLRP3 inflammasome-related protein expression, indicating successful activation of the cGAS–STING–NLRP3 axis in this *in vitro* model. Notably, pretreatment with either THF-serum or RU.521 markedly attenuated this upregulation (**Figures 6E, F**), suggesting that THF effectively suppresses cGAMP-induced inflammasome activation.

Regarding pyroptosis, TUNEL and caspase-1 immunofluorescence co-staining revealed that cGAMP stimulation significantly increased the number of TUNEL and caspase-1 double-positive NMCMs, whereas RU.521 or THF-serum pretreatment substantially reduced the double-positive cell population (**Figures 6G, H**), further confirming that THF effectively inhibits cGAS–STING-mediated pyroptosis.

Moreover, molecular docking provided structural evidence for the mechanism of action of THF. The major active components of THF—Berberine, Coptisine, and Palmatine may all form complexes with the active sites of human cGAS through non-covalent interactions, potentially inhibiting its catalytic activity. Berberine exhibited the highest binding affinity (–8.8 kcal/mol), primarily through hydrogen bonding with the key residue PHE-379. Coptisine exhibited a binding energy of –8.2 kcal/mol and interacted with SER-492, whereas Palmatine, with a binding energy of –7.5 kcal/mol, did not form obvious hydrogen bonds but may stably occupy the active pocket through hydrophobic interactions or π–π stacking. These findings not only elucidate the precise interaction patterns between the active compounds and the target protein but also provide a theoretical basis for THF as a potential cGAS inhibitor (**Figures 6I–K**).

In summary, these *in vitro* results demonstrate that THF attenuates NLRP3 inflammasome activation and cardiomyocyte pyroptosis by suppressing the overactivation of the cGAS–STING inflammatory pathway, thereby exerting significant anti-inflammatory and cardioprotective effects.

## 4 Discussion

**Figure 7:**
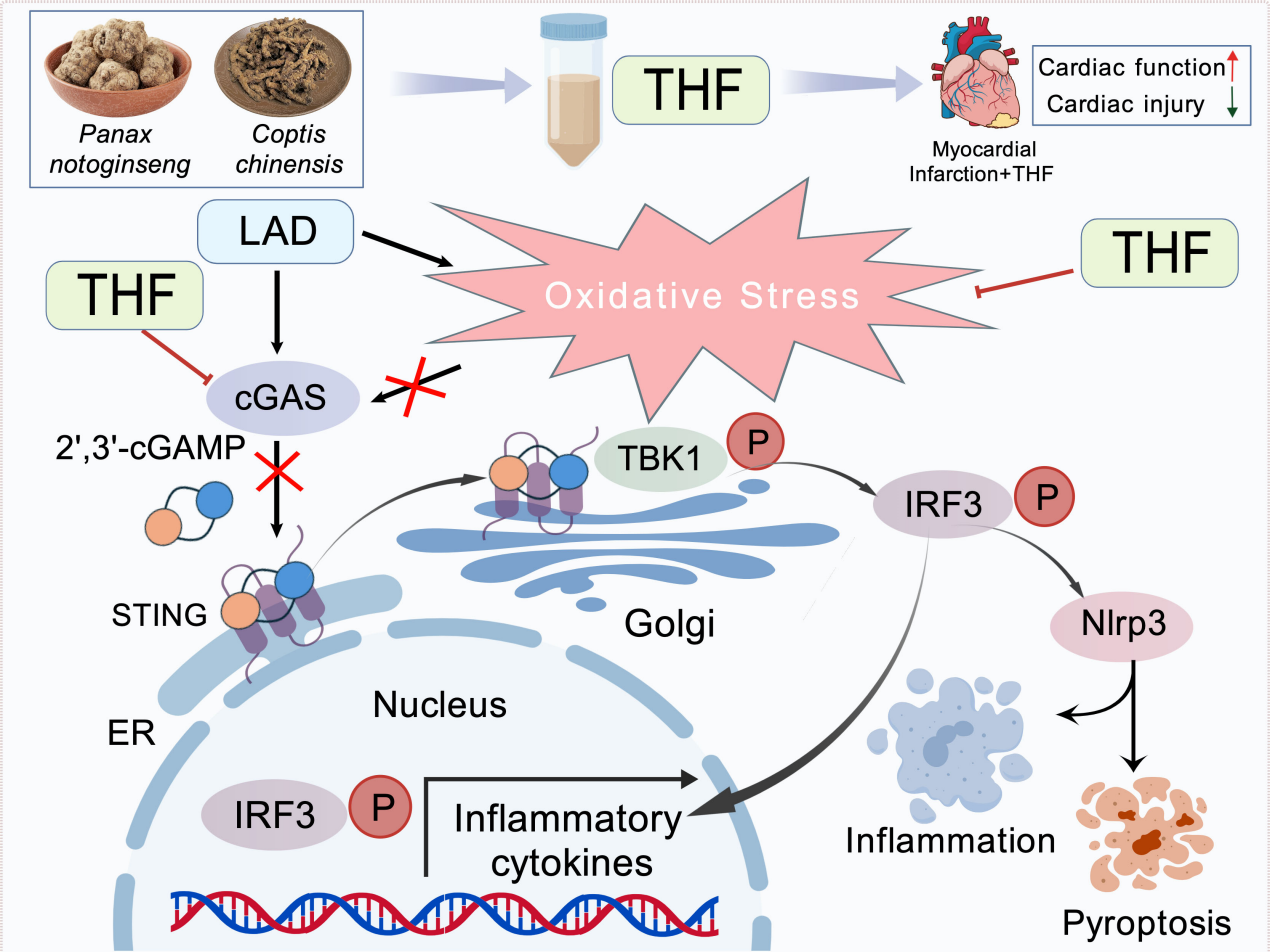
MI induced by LAD artery ligation triggers cardiac oxidative stress, activating the cytosolic DNA sensor cGAS. This promotes synthesis of 2’,3’-cGAMP, which binds STING on ER/Golgi membranes. STING recruits TBK1 to phosphorylate IRF3. Concurrently, STING signaling activates the NLRP3, leading to caspase-1-mediated pyroptosis. THF treatment potently inhibits the cGAS-STING axis by suppressing cGAS activation and downstream TBK1/IRF3 signaling. This attenuates NLRP3 inflammasome assembly, caspase-1 cleavage, and pyroptotic cytokine secretion, ultimately mitigating cardiac inflammation and tissue injury.

This study demonstrates that THF exerts cardioprotective effects by reducing oxidative stress, modulating the cGAS–STING–NLRP3 signaling axis, and inhibiting cardiomyocyte pyroptosis (**Figure 7**). These findings provide a theoretical basis for THF as a potential therapeutic agent for MI and further elucidate its molecular mechanisms.

MI is a severe cardiovascular disease primarily caused by the sudden occlusion of coronary arteries—often due to atherosclerotic plaque rupture and thrombosis—resulting in prolonged myocardial ischemia (57). Following MI, injury to cardiomyocytes triggers excessive production of ROS through mitochondrial oxidative phosphorylation, which in turn facilitates rapid infiltration of pro-inflammatory immune cells into the ischemic myocardium. This amplified inflammatory response exacerbates myocardial ischemic injury and ultimately contributes to the progression toward heart failure (58).

Earlier studies indicate that cGAS is essential for innate immune responses, functioning by sensing cytoplasmic DNA and triggering activation of the STING signaling cascade. Activation of STING further stimulates downstream signaling pathways, including NF-κB and TBK1–IRF3, ultimately leading to NLRP3 inflammasome-mediated pyroptosis (59). Importantly, NLRP3 inflammasome activation drives collagen deposition and plays a part in developing hypertrophy and fibrosis. This pathological remodeling is mediated by the secretion of pro-inflammatory cytokines directly into the heart’s matrix (60–62). Therefore, targeting the cGAS–STING–NLRP3 signaling pathway may be a promising therapeutic strategy for treating immunoinflammatory injury resulting from myocardial ischemia.

TCM has a history of use in China spanning thousands of years, with numerous herbal formulations demonstrating significant anti-inflammatory and antioxidant effects (63). In recent years, growing evidence has shown that TCM can modulate myocardial energy metabolism, inhibit cardiomyocyte apoptosis, enhance endothelial function, and reduce cardiac remodeling. These mechanisms offer promising therapeutic avenues for ischemic cardiovascular diseases such as MI and HF (64). Against this background, Fufang Zhenzhu Tiaozhi Capsules (FTZ) were developed by Professor Guo Jiao’s team at Guangdong Pharmaceutical University, based on decades of clinical experience and traditional Chinese medicine syndrome-differentiation theory. Comprising eight herbs—including Ligustrum lucidum, Citrus medica, *Panax notoginseng*, *Coptis chinensis*, and Salvia miltiorrhiza—FTZ exerts comprehensive lipid-regulating and hypoglycemic effects. In 2024, FTZ was recognized by the Guangdong Provincial Drug Administration as part of the second batch of Lingnan formulas and incorporated into the Guangdong provincial medical insurance system (65). Modern pharmacological studies have demonstrated that FTZ alleviates angiotensin II (Ang II)-induced oxidative stress injury in neonatal cardiomyocytes via antioxidant pathways (66), while also inhibiting activation of the NLRP3 inflammasome and its downstream pyroptotic signaling, thereby slowing the progression of atherosclerosis (67). Subsequently, Professor Guo’s team systematically screened the core active components of FTZ and, based on this, developed a novel, more defined, and simplified formulation—THF. THF is a patented TCM formula consisting of *Panax notoginseng* and *Coptis chinensis* in a 1:1 ratio, developed based on Professor Guo Jiao’s “Tiaogan Qishu Huazhuo” theory for the treatment of glycolipid metabolic disorders. It aims to improve myocardial perfusion, remove metabolic waste, and optimize cardiac metabolism (29). *Panax notoginseng* has traditionally been used to promote blood circulation, stop bleeding, relieve pain, and reduce swelling, particularly in cardiovascular diseases (20, 68). Its main active compounds, notoginsenosides, can stimulate cardiomyocyte autophagy via AMPK and CaMKII phosphorylation, providing cardio protection consistent with traditional use (24). *Coptis chinensis* has traditionally been used to clear heat, detoxify, and alleviate palpitations, and it is widely applied in the treatment of cardiovascular diseases such as coronary heart disease and heart HF (26, 69). Studies have shown that its main active component, Coptisine, exhibits anti-inflammatory and cardioprotective effects, and can ameliorate myocardial infarction–associated damage (70). THF retains the traditional rationale of promoting circulation and resolving stasis while offering a simplified, evidence-based cardioprotective formulation.

Several studies have reported that THF shows marked therapeutic effects in liver diseases. THF exerts its effects by activating the “Lactobacillus–5-MIAA–Nrf2” pathway to regulate the gut microbiota in mice, thereby reducing oxidative stress in NAFLD (30). Moreover, THF also alleviated hepatic injury and inflammation by inhibiting the CCL2–CCR2 axis (32). Furthermore, THF eases glucose intolerance and hepatic steatosis through the activation of PI3K and LDL-R pathways (29). Similarly, in T2DM mice, it ameliorates impairments in glucose and lipid metabolism by promoting mitochondrial activity within adipose tissue via activation of the AMPK/MICU1 pathway (71, 72). Given the significant therapeutic effects of THF in various metabolism- and inflammation-related diseases, we hypothesize that it may exert protective effects in MI through similar anti-inflammatory, antioxidant, and metabolic regulatory mechanisms. To validate the cardioprotective effects of THF, we established a mouse model of MI via ligation of the LAD coronary artery, followed by continuous oral administration of THF for 28 days. During the acute phase (postoperative day 3), MI mice exhibited significant cardiac dysfunction, including reduced EF and FS, as well as increased LVIDd and LVIDs. Notably, only three days of THF treatment significantly improved EF and FS, with trends toward decreased LVIDd and LVIDs, indicating effective mitigation of acute cardiac dysfunction.

In the chronic remodeling phase (postoperative day 28), MI mice continued to display impaired cardiac function and ventricular dilation. THF treatment markedly improved these parameters, suggesting its role in promoting long-term cardiac recovery and attenuating adverse remodeling. Histological analyses (WGA and HE staining) revealed that THF alleviated myocardial fiber disarray, widened intercellular spaces, and extensive inflammatory cell infiltration. Correspondingly, THF downregulated mRNA expression of hypertrophic markers, further suppressing pathological remodeling.

Given the central role of inflammation in MI pathophysiology—peaking during the acute phase and driving subsequent remodeling—we assessed serum inflammatory cytokines. THF significantly reduced inflammatory factor levels in both acute and chronic phases and ameliorated myocardial tissue inflammation, suggesting that it not only suppresses acute inflammatory responses (reducing mediator release and immune cell recruitment) but also modulates chronic immune inflammation, thereby improving the myocardial microenvironment and mitigating adverse ventricular remodeling.

Mechanistically, integration of network pharmacology, blood microarray data from MI patients, and mouse cardiac transcriptomics highlighted the cGAS-STING signaling pathway as a key mediator of THF’s cardioprotective effects. *In vivo* validation experiments (Western blot and qPCR) confirmed that MI significantly upregulated cardiac proteins and mRNA associated with the cGAS-STING pathway, whereas THF effectively suppressed their activation and concurrently reduced markers of myocardial injury (LDH activity) and oxidative stress (MDA and 8-OHdG levels).

Downstream analysis revealed that MI activated the NLRP3 inflammasome pathway, as evidenced by elevated NLRP3, Cleaved-caspase-1, and the pyroptotic executor GSDMD-N. THF significantly inhibited their expression, suggesting suppression of inflammasome-mediated pyroptosis via blockade of the cGAS-STING/NLRP3 axis. This effect was further validated in an H_2_O_2_-induced cardiomyocyte hypoxia/oxidative stress model: THF serum treatment improved cell viability and function while inhibiting cGAS-STING activation and downstream NLRP3 inflammasome assembly and pyroptosis (reduced caspase-1, GSDMD-N, and TUNEL-positive cells). Both cGAS inhibition (RU.521) and THF pre-treatment suppressed pyroptosis, and even under cGAMP stimulation, THF effectively blocked NLRP3 inflammasome-mediated pyroptosis, confirming its upstream action on the cGAS-STING pathway.

Moreover, from the perspective of active components, molecular docking analysis demonstrated that the key constituents of THF—berberine, coptisine, and palmatine—exhibit strong binding affinities to human cGAS, supporting a direct molecular interaction through which THF may modulate the cGAS–STING signaling axis.

In summary, THF significantly improves post-MI cardiac function and ventricular remodeling through multi-target effects, including suppression of oxidative stress, modulation of immune-inflammatory responses, and attenuation of pyroptotic cell death. A central mechanism contributing to these cardioprotective effects appears to involve the suppression of the cGAS–STING pathway and downstream NLRP3 inflammasome activation.

The convergent evidence from multi-omics, *in vivo* and *in vitro* models, and pharmacological intervention robustly positions the cGAS–STING–NLRP3 pathway as a key mechanism associated with THF’s therapeutic action. While this strong association provides a compelling mechanistic hypothesis, the current experimental approach cannot conclusively prove that the pathway is the exclusive or direct target of THF. Future studies employing genetic perturbations will be essential to validate causal necessity and to dissect whether THF modulates this axis directly or via upstream events. Notably, network pharmacology and transcriptomic analyses were first used as supportive, hypothesis-generating tools to identify overarching pathological themes such as chronic inflammation and oxidative stress. Subsequent protein-level assays and pharmacological interventions in animal and cellular models confirmed that the cGAS–STING–NLRP3 axis plays a central role in THF-mediated cardioprotection. THF significantly alleviated myocardial oxidative stress and DNA damage, and improved mitochondrial structure and function. Given that oxidative stress and mitochondrial DNA damage can trigger endogenous DNA leakage and activate cGAS–STING, THF may partially modulate this pathway indirectly by restoring redox balance and mitochondrial homeostasis. Molecular docking suggested potential interactions between several active compounds and cGAS, but these predictions require direct biochemical validation, such as cGAS enzymatic activity, STING oligomerization, or downstream signaling assays. Overall, this study combines computational tools to generate mechanistic hypotheses with targeted experiments to validate them, providing multi-level evidence for THF’s cardioprotective mechanisms.

Regarding the spectrum of cell death, THF treatment markedly downregulates inflammasome and pyroptosis-associated markers, including NLRP3, cleaved caspase-1, and GSDMD-N, and immunostaining indicates a reduction in inflammasome-associated cell death phenotypes, suggesting that inflammatory cell death is modulated in myocardial infarction. The cGAS–STING pathway can not only mediate pyroptosis through NLRP3 but also regulate apoptosis-related genes via downstream TBK1–IRF3 and NF-κB signaling, potentially enhancing caspase-dependent cell death under certain conditions. Therefore, relying solely on changes in NLRP3, cleaved caspase-1, and GSDMD-N is insufficient to strictly define the observed cell death as pyroptosis. Although TUNEL and caspase-1 colocalization reflects the occurrence of inflammatory cell death, it cannot fully distinguish pyroptosis, apoptosis, or other forms of DNA-fragmentation-associated cell death. Furthermore, the generation and release of mature IL-1β and IL-18, which are key functional endpoints of inflammasome-mediated pyroptosis, were not directly assessed.

Based on these limitations, the current evidence more strongly supports an inflammasome-associated, pyroptosis-like cell death phenotype modulated by THF, rather than an exclusive definition of pyroptosis. Combining cardiomyocyte-specific genetic interventions targeting cGAS/STING or functional validation of individual active compounds would allow quantification of the relative contributions of different pathways to THF-mediated cardioprotection. In addition, correlating key targets with THF efficacy in clinical samples could help to further evaluate its translational potential.

## Data Availability

The gene expression dataset analyzed in this study has been deposited in the Gene Expression Omnibus (GEO) database under accession number GSE66360 (https://www.ncbi.nlm.nih.gov/geo/query/acc.cgi?acc=GSE66360). The original contributions presented in the study are included in the article/**Supplementary Material**. Further inquiries can be directed to the corresponding authors.
