# Supplementary material for: Tianhuang formula attenuates cardiomyocyte pyroptosis in myocardial infarction by suppressing oxidative stress and the cGAS–STING–NLRP3 axis
Source: Front Immunol. 2026 Feb 20;17:1761299. doi: 10.3389/fimmu.2026.1761299 (PMC12965622; doi:10.3389/fimmu.2026.1761299)
Supplement: Supplementary file 1 [file DataSheet1.pdf]

广东药科大学实验动物伦理审查意见表

申请人填写处

实验 / 课题名称：
田黄方对心肌梗死小鼠的改善作用

申请人：陈怡凡

实验动物伦理委员会填写处

受理编号：gdpulacspf2022156

一、具体审查内容

评审条目	评审说明	是	否
1、动物实验的必要性	实验方案是否科学、合理	✓	
	是否不能用非动物模型模拟动物实验	✓	
	是否有体外实验作为基础	✓	
2、使用实验动物种类的合理性	是否没有更小型的实验动物可以替代	✓	
	选择的实验动物种类是必需的	✓	
3、使用实验动物数量的合理性	在符合统计学要求的情况下，是否使用最少数量的实验动物	✓	
4、手术方案是否符合伦理要求	手术前是否实施动物麻醉	✓	
	选择的麻醉药物和麻醉途径是否合理	✓	
	在满足实验要求的情况下手术方式是否可以将动物的痛苦减到最低	✓	
5、动物护理措施是否符合伦理要求	手术后采用的动物护理措施是否能将动物的痛苦减到最低	✓	
	是否给予最好的营养和饲养环境	✓	
6、实验周期合理性	在满足实验要求的情况下，实验周期是否最短	✓	
7、实验结束后动物的处理是否符合伦理要求	是否采用将痛苦减到最低的处死方式处理实验结束后的动物，如麻醉处死（安乐死术）	✓	
	实验动物尸体、标本、废弃物的处理是否符合无害化处理方案	✓	

二、审查结果

☑ 同意　　　☐ 不同意　　　☐ 待修正后再审

审查者签名：[illegible]　　2023 年 6 月 30 日

伦理委员会主任或副主任签名：[illegible]　　2023 年 6 月 30 日

广东药科大学实验动物伦理委员会（公章）

注：以上条件要全部符合，才视为符合动物伦理要求，否则，需要调整实验方案。

扫描全能王
3亿人都在用的扫描App
