# Supplementary material for: Tianhuang formula attenuates cardiomyocyte pyroptosis in myocardial infarction by suppressing oxidative stress and the cGAS–STING–NLRP3 axis
Source: Front Immunol. 2026 Feb 20;17:1761299. doi: 10.3389/fimmu.2026.1761299 (PMC12965622; doi:10.3389/fimmu.2026.1761299)
Supplement: Supplementary file 2 [file DataSheet2.docx]

**Experimental Animal Ethics Review Opinion Form of Guangdong Pharmaceutical University**

^1^

**Project/Subject Name:** Effect of Tianhuang Prescription on Myocardial Infarction in Mice **Applicant:** Chen Yifan **Application Number:** gdpulacspf2022256

**I. Specific Review Content**

| **Review Item** | **Review Description** | **Yes/No** |
| --- | --- | --- |
| 1. Necessity of animal experiments | Is the experimental design scientific and reasonable? Can non-animal models simulate the experiment? Is there a basis of in vitro experiments? | Yes |
| 2. Rationality of animal species | Is there no smaller experimental animal that can replace it? Is the selected species essential? | Yes |
| 3. Rationality of animal quantity | Is the minimum number of animals used while meeting statistical requirements? | Yes |
| 4. Ethical requirements of surgical protocol | Is anesthesia administered before surgery? Are the anesthetic drugs and routes reasonable? Does the surgical method minimize pain? | Yes |
| 5. Ethical requirements of animal care | Do post-operative care measures minimize pain? Are the best nutrition and breeding environment provided? | Yes |
| 6. Rationality of experimental cycle | Is the experimental cycle the shortest possible while meeting experimental requirements? | Yes |
| 7. Post-experiment animal disposal | Is a method used to minimize pain (e.g., euthanasia)? Does the disposal of carcasses and waste comply with harmless treatment protocols? | Yes |
