# Supplementary material for: Tianhuang formula attenuates cardiomyocyte pyroptosis in myocardial infarction by suppressing oxidative stress and the cGAS–STING–NLRP3 axis
Source: Front Immunol. 2026 Feb 20;17:1761299. doi: 10.3389/fimmu.2026.1761299 (PMC12965622; doi:10.3389/fimmu.2026.1761299)
Supplement: Supplementary file 3 [file DataSheet3.zip › GEO66360/GSE66360τü1⁄2σ▒▒σ¢╛-τâ¡σ¢╛Σ╗úτáü.docx]

## ===============================

## 0. 加载所需 R 包

## ===============================

library(limma)

library(GEOquery)

library(stringr)

library(ggplot2)

## ===============================

## 1. 下载并读取 GSE66360 表达矩阵

## ===============================

gset <- getGEO(

"GSE66360",

destdir = ".",

AnnotGPL = FALSE,

getGPL = FALSE

)

expr <- exprs(gset[[1]]) # 表达矩阵

## ===============================

## 2. 判断是否需要 log2 转换

## ===============================

summary(expr)

if (max(expr, na.rm = TRUE) > 1000) {

expr <- log2(expr + 1)

message("表达矩阵已进行 log2 转换")

}

## ===============================

## 3. 样本信息与分组

## ===============================

pdata <- pData(gset[[1]])

table(pdata$title)

group_list <- ifelse(

str_detect(trimws(pdata$title), "Myocardial Infarction"),

"MI",

"normal"

)

group_list <- factor(group_list, levels = c("normal", "MI"))

table(group_list)

## ===============================

## 4. 读取 GPL570 注释文件

## ===============================

gpl <- read.table(

"GPL570.txt",

header = TRUE,

sep = "\t",

quote = "",

stringsAsFactors = FALSE

)

## 保留有基因名的探针

gpl <- gpl[gpl$Gene.Symbol != "" & !is.na(gpl$Gene.Symbol), ]

## 处理 Gene.Symbol（保留 /// 前第一个）

gpl$Gene.Symbol <- sapply(

str_split(gpl$Gene.Symbol, "///"),

function(x) str_replace_all(x[1], " ", "")

)

## 去除重复基因

gpl <- gpl[!duplicated(gpl$Gene.Symbol), ]

## ===============================

## 5. 注释合并表达矩阵

## ===============================

expr_annot <- merge(

gpl[, c("ID", "Gene.Symbol")],

expr,

by.x = "ID",

by.y = "row.names"

)

rownames(expr_annot) <- expr_annot$Gene.Symbol

expr_annot <- expr_annot[, -(1:2)]

## ===============================

## 6. 表达矩阵标准化（quantile）

## ===============================

expr_norm <- normalizeBetweenArrays(

as.matrix(expr_annot),

method = "quantile"

)

## ===============================

## 7. 构建设计矩阵 & limma 分析

## ===============================

design <- model.matrix(~ 0 + group_list)

colnames(design) <- levels(group_list)

fit <- lmFit(expr_norm, design)

contrast.matrix <- makeContrasts(

MI_vs_normal = MI - normal,

levels = design

)

fit2 <- contrasts.fit(fit, contrast.matrix)

fit2 <- eBayes(fit2)

## ===============================

## 8. 差异基因筛选

## ===============================

results <- topTable(

fit2,

adjust.method = "fdr",

number = Inf

)

sig_genes <- results[

results$adj.P.Val < 0.05 & abs(results$logFC) > 0.5,

]

message("显著差异基因数：", nrow(sig_genes))

write.table(

sig_genes,

file = "significant_genes.txt",

sep = "\t",

quote = FALSE,

col.names = NA

)

## ===============================

## 9. 火山图数据整理

## ===============================

results$color <- ifelse(

results$adj.P.Val < 0.05 & results$logFC > 0.5,

"Up",

ifelse(

results$adj.P.Val < 0.05 & results$logFC < -0.5,

"Down",

"Not significant"

)

)

## ===============================

## 10. 绘制火山图

## ===============================

volcano_plot <- ggplot(

results,

aes(x = logFC, y = -log10(adj.P.Val))

) +

geom_point(

data = results[results$color == "Up", ],

aes(color = "Up"),

shape = 2,

size = 3

) +

geom_point(

data = results[results$color == "Down", ],

aes(color = "Down"),

shape = 6,

size = 3

) +

geom_point(

data = results[results$color == "Not significant", ],

aes(color = "Not significant"),

size = 2

) +

scale_color_manual(

values = c(

"Up" = "orangered",

"Down" = "skyblue",

"Not significant" = "gray"

),

name = "Regulated"

) +

theme_minimal() +

xlab("log2 Fold Change") +

ylab("-log10(FDR)") +

geom_hline(

yintercept = -log10(0.05),

linetype = "dashed"

) +

geom_vline(

xintercept = c(-0.5, 0.5),

linetype = "dashed"

) +

scale_x_continuous(

limits = c(-4, 4),

breaks = c(-4, -2, 0, 2, 4)

) +

ylim(0, max(-log10(results$adj.P.Val), na.rm = TRUE))

## ===============================

## 11. 导出火山图

## ===============================

ggsave(

filename = "volcano_plot.tiff",

plot = volcano_plot,

width = 10,

height = 6,

dpi = 300

)

print(volcano_plot)

message("火山图已成功导出：volcano_plot.tiff")

# 颜色：低表达（蓝）- 中间（白）- 高表达（红）

heatmap_colors <- colorRampPalette(

c("#2C7BB6", "white", "#D7191C")

)(100)

# 固定 Z-score 映射区间（与你图中 -4 ~ 4 一致）

bk <- seq(-4, 4, length.out = 101)

library(pheatmap)

## 1. Top30 基因（按 FDR）

top30_genes <- rownames(sig_genes)[

order(sig_genes$adj.P.Val)

][1:30]

## 2. 表达矩阵

heatmap_mat <- expr_norm[top30_genes, ]

## 3. Z-score（按基因）

heatmap_mat_scaled <- t(scale(t(heatmap_mat)))

## 4. 样本分组注释

annotation_col <- data.frame(

Group = group_list

)

rownames(annotation_col) <- colnames(heatmap_mat_scaled)

annotation_colors <- list(

Group = c(

normal = "#66C2A5", # 和你图里一致的青绿

MI = "#FC8D62" # 橙红

)

)

## 5. 颜色（与示例图一致）

heatmap_colors <- colorRampPalette(

c("#2C7BB6", "white", "#D7191C")

)(100)

bk <- seq(-4, 4, length.out = 101)

## 6. 绘制热图

pheatmap(

heatmap_mat_scaled,

color = heatmap_colors,

breaks = bk,

annotation_col = annotation_col,

annotation_colors = annotation_colors,

cluster_rows = TRUE,

cluster_cols = TRUE,

show_colnames = FALSE,

show_rownames = TRUE,

border_color = NA,

fontsize_row = 9,

main = "Top 30 Significant Differentially Expressed Genes",

filename = "Top30_DEG_heatmap.tiff",

width = 10,

height = 8

)
