# Supplementary material for: Tianhuang formula attenuates cardiomyocyte pyroptosis in myocardial infarction by suppressing oxidative stress and the cGAS–STING–NLRP3 axis
Source: Front Immunol. 2026 Feb 20;17:1761299. doi: 10.3389/fimmu.2026.1761299 (PMC12965622; doi:10.3389/fimmu.2026.1761299)
Supplement: Supplementary file 4 [file DataSheet4.zip › WB-Raw data/Figure S1 WB.pptx]

## Slide 1
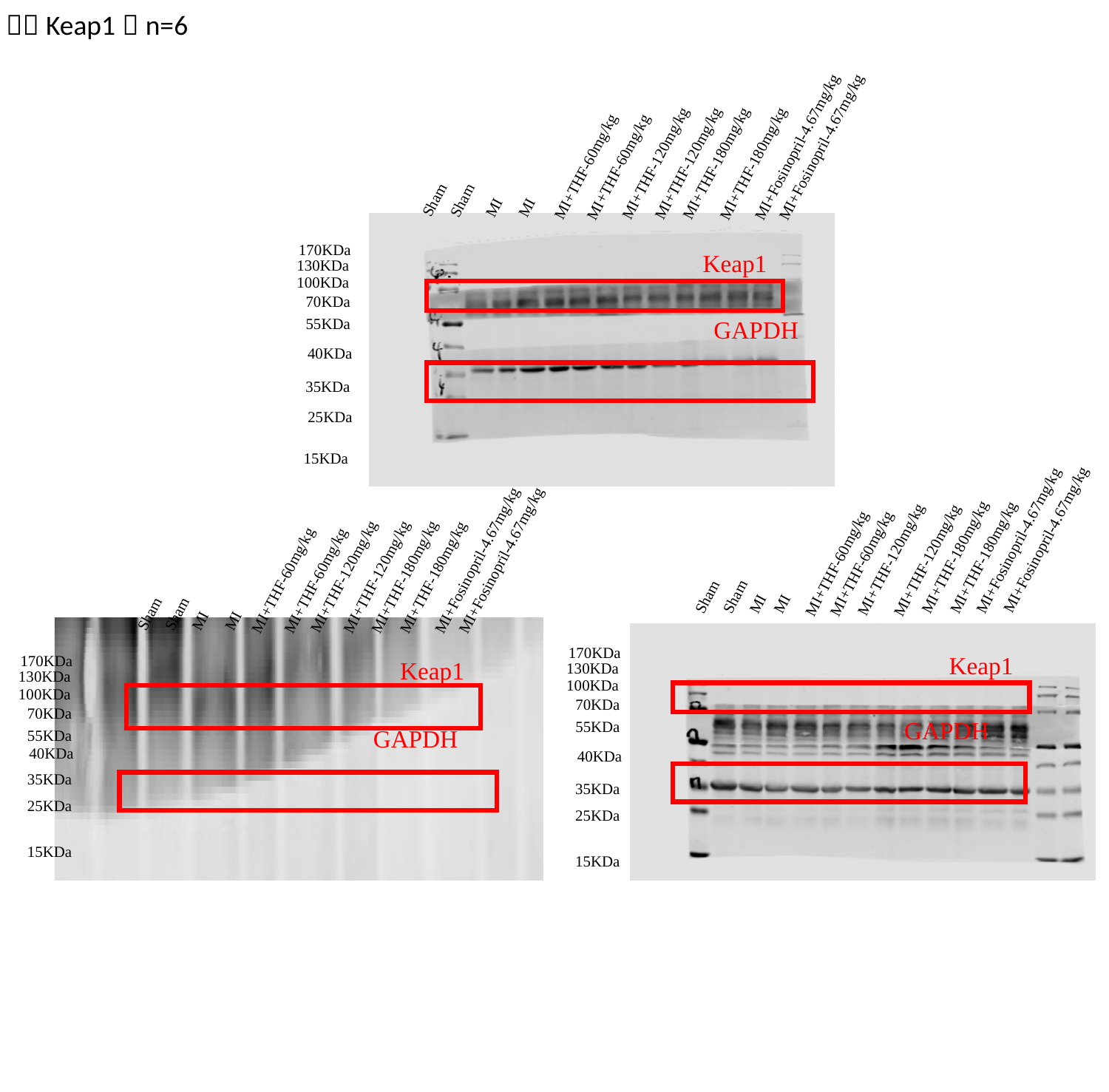

组织Keap1，n=6
MI+Fosinopril-4.67mg/kg
MI+Fosinopril-4.67mg/kg
MI+THF-60mg/kg
MI+THF-180mg/kg
MI+THF-120mg/kg
MI+THF-60mg/kg
MI+THF-180mg/kg
MI+THF-120mg/kg
Sham
Sham
MI
MI
170KDa
Keap1
130KDa
100KDa
70KDa
55KDa
GAPDH
40KDa
35KDa
25KDa
15KDa
MI+Fosinopril-4.67mg/kg
MI+Fosinopril-4.67mg/kg
MI+THF-180mg/kg
MI+THF-60mg/kg
MI+THF-180mg/kg
MI+THF-120mg/kg
MI+THF-60mg/kg
MI+THF-120mg/kg
MI+Fosinopril-4.67mg/kg
MI+Fosinopril-4.67mg/kg
MI+THF-60mg/kg
MI+THF-180mg/kg
MI+THF-120mg/kg
MI+THF-60mg/kg
MI+THF-180mg/kg
MI+THF-120mg/kg
Sham
MI
MI
Sham
Sham
Sham
MI
MI
170KDa
Keap1
170KDa
Keap1
130KDa
130KDa
100KDa
100KDa
70KDa
70KDa
GAPDH
55KDa
GAPDH
55KDa
40KDa
40KDa
35KDa
35KDa
25KDa
25KDa
15KDa
15KDa

## Slide 2
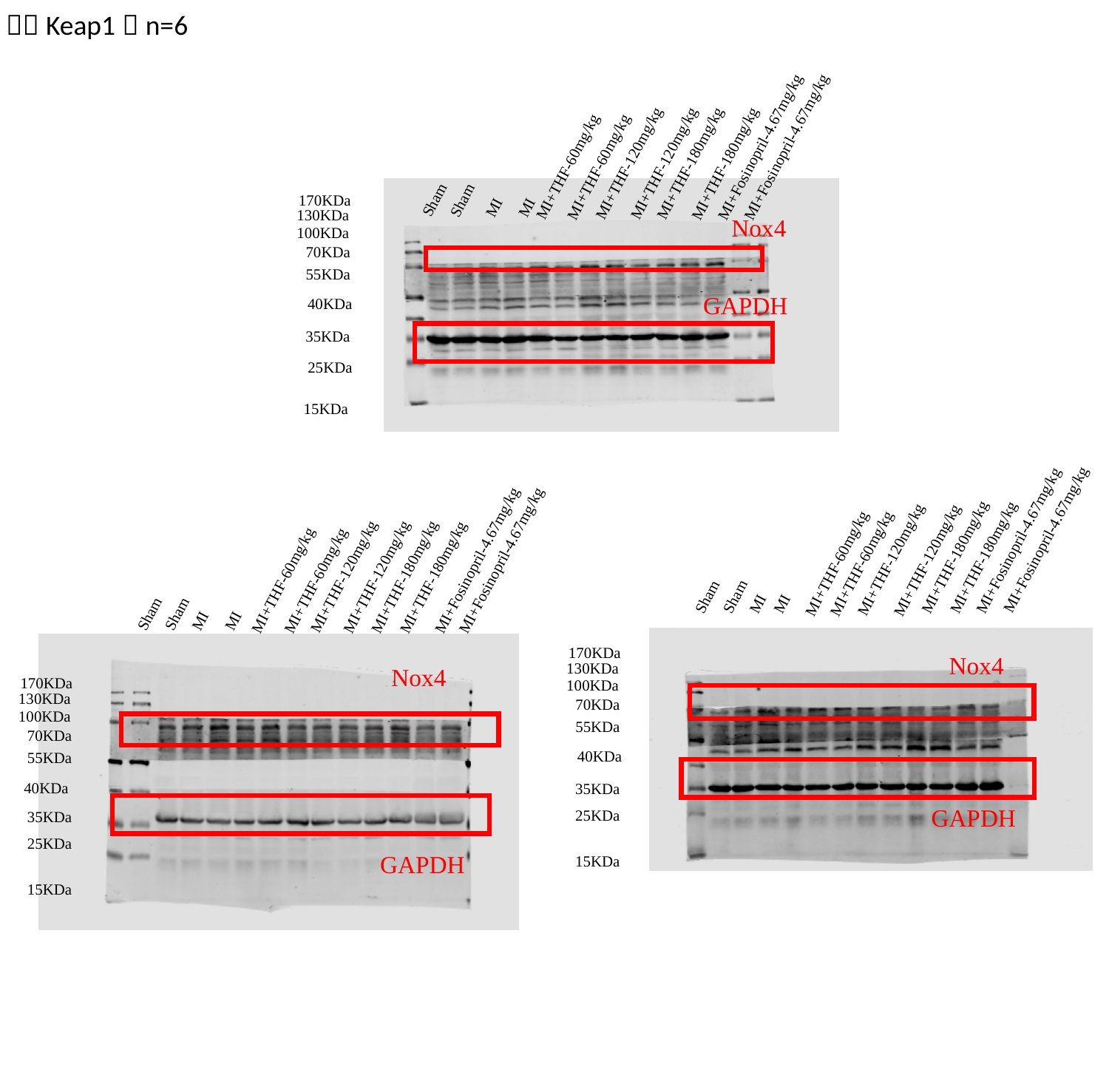

组织Keap1，n=6
MI+Fosinopril-4.67mg/kg
MI+Fosinopril-4.67mg/kg
MI+THF-60mg/kg
MI+THF-180mg/kg
MI+THF-120mg/kg
MI+THF-60mg/kg
MI+THF-180mg/kg
MI+THF-120mg/kg
Sham
Sham
MI
MI
170KDa
130KDa
Nox4
100KDa
70KDa
55KDa
GAPDH
40KDa
35KDa
25KDa
15KDa
MI+Fosinopril-4.67mg/kg
MI+Fosinopril-4.67mg/kg
MI+THF-180mg/kg
MI+THF-60mg/kg
MI+THF-180mg/kg
MI+THF-120mg/kg
MI+THF-60mg/kg
MI+THF-120mg/kg
MI+Fosinopril-4.67mg/kg
MI+Fosinopril-4.67mg/kg
MI+THF-60mg/kg
MI+THF-180mg/kg
MI+THF-120mg/kg
MI+THF-60mg/kg
MI+THF-180mg/kg
MI+THF-120mg/kg
Sham
MI
MI
Sham
Sham
Sham
MI
MI
170KDa
Nox4
130KDa
Nox4
170KDa
100KDa
130KDa
70KDa
100KDa
55KDa
70KDa
40KDa
55KDa
40KDa
35KDa
GAPDH
25KDa
35KDa
25KDa
GAPDH
15KDa
15KDa

## Slide 3
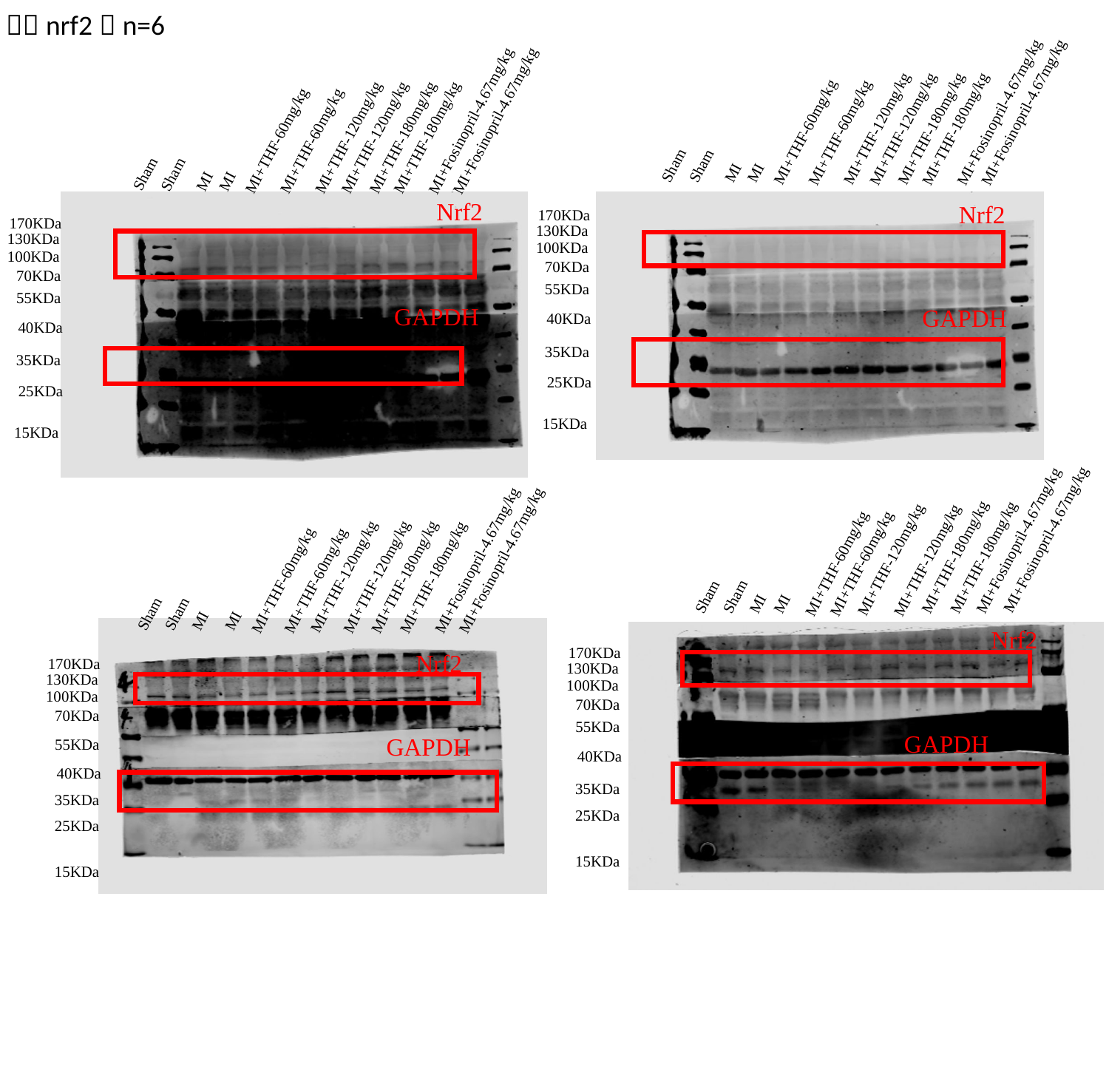

组织nrf2，n=6
MI+Fosinopril-4.67mg/kg
MI+Fosinopril-4.67mg/kg
MI+THF-60mg/kg
MI+THF-180mg/kg
MI+THF-120mg/kg
MI+THF-60mg/kg
MI+THF-180mg/kg
MI+Fosinopril-4.67mg/kg
MI+Fosinopril-4.67mg/kg
MI+THF-60mg/kg
MI+THF-120mg/kg
MI+THF-180mg/kg
MI+THF-120mg/kg
MI+THF-60mg/kg
MI+THF-180mg/kg
MI+THF-120mg/kg
Sham
Sham
MI
MI
Sham
Sham
MI
MI
Nrf2
Nrf2
170KDa
170KDa
130KDa
130KDa
100KDa
100KDa
70KDa
70KDa
55KDa
55KDa
GAPDH
GAPDH
40KDa
40KDa
35KDa
35KDa
25KDa
25KDa
15KDa
15KDa
MI+Fosinopril-4.67mg/kg
MI+Fosinopril-4.67mg/kg
MI+THF-180mg/kg
MI+THF-60mg/kg
MI+THF-180mg/kg
MI+THF-120mg/kg
MI+THF-60mg/kg
MI+THF-120mg/kg
MI+Fosinopril-4.67mg/kg
MI+Fosinopril-4.67mg/kg
MI+THF-60mg/kg
MI+THF-180mg/kg
MI+THF-120mg/kg
MI+THF-60mg/kg
MI+THF-180mg/kg
MI+THF-120mg/kg
Sham
MI
MI
Sham
Sham
Sham
MI
MI
Nrf2
170KDa
Nrf2
170KDa
130KDa
130KDa
100KDa
100KDa
70KDa
70KDa
55KDa
GAPDH
GAPDH
55KDa
40KDa
40KDa
35KDa
35KDa
25KDa
25KDa
15KDa
15KDa
