# Supplementary material for: Tianhuang formula attenuates cardiomyocyte pyroptosis in myocardial infarction by suppressing oxidative stress and the cGAS–STING–NLRP3 axis
Source: Front Immunol. 2026 Feb 20;17:1761299. doi: 10.3389/fimmu.2026.1761299 (PMC12965622; doi:10.3389/fimmu.2026.1761299)
Supplement: Supplementary file 4 [file DataSheet4.zip › WB-Raw data/Figure 4G WB.pptx]

## Slide 1
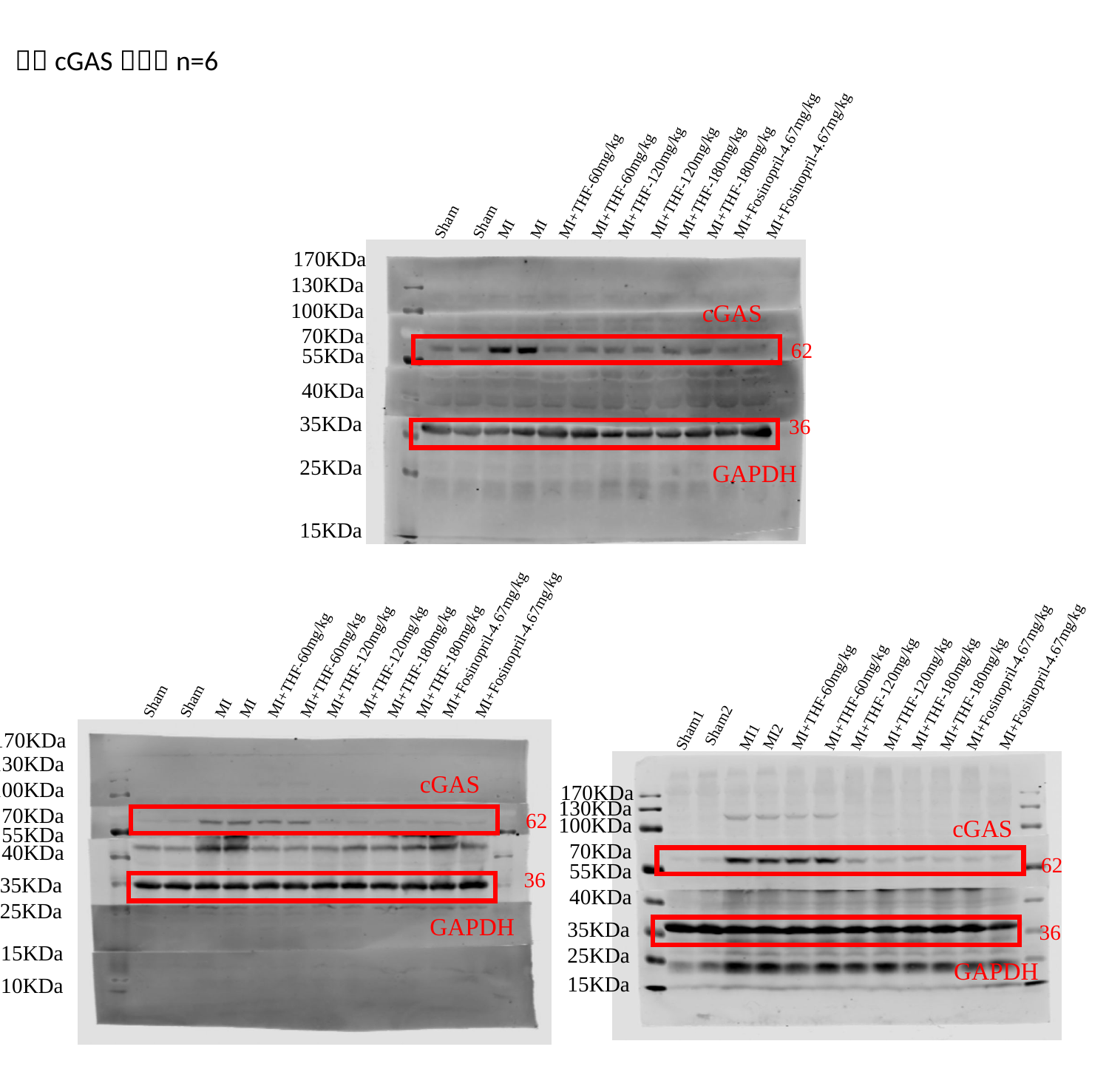

组织cGAS蛋白，n=6
MI+Fosinopril-4.67mg/kg
MI+Fosinopril-4.67mg/kg
MI+THF-60mg/kg
MI+THF-180mg/kg
MI+THF-120mg/kg
MI+THF-60mg/kg
MI+THF-180mg/kg
MI+THF-120mg/kg
Sham
MI
MI
Sham
170KDa
130KDa
100KDa
cGAS
70KDa
62
55KDa
40KDa
35KDa
36
25KDa
GAPDH
15KDa
MI+Fosinopril-4.67mg/kg
MI+Fosinopril-4.67mg/kg
MI+THF-60mg/kg
MI+THF-180mg/kg
MI+THF-120mg/kg
MI+THF-60mg/kg
MI+THF-180mg/kg
MI+THF-120mg/kg
MI+Fosinopril-4.67mg/kg
MI+Fosinopril-4.67mg/kg
MI+THF-60mg/kg
MI+THF-180mg/kg
MI+THF-120mg/kg
MI+THF-60mg/kg
MI+THF-180mg/kg
MI+THF-120mg/kg
Sham
Sham
MI
MI
Sham2
MI2
Sham1
MI1
170KDa
130KDa
cGAS
100KDa
170KDa
130KDa
70KDa
62
100KDa
cGAS
55KDa
70KDa
40KDa
62
55KDa
36
35KDa
40KDa
25KDa
GAPDH
35KDa
36
15KDa
25KDa
GAPDH
15KDa
10KDa

## Slide 2
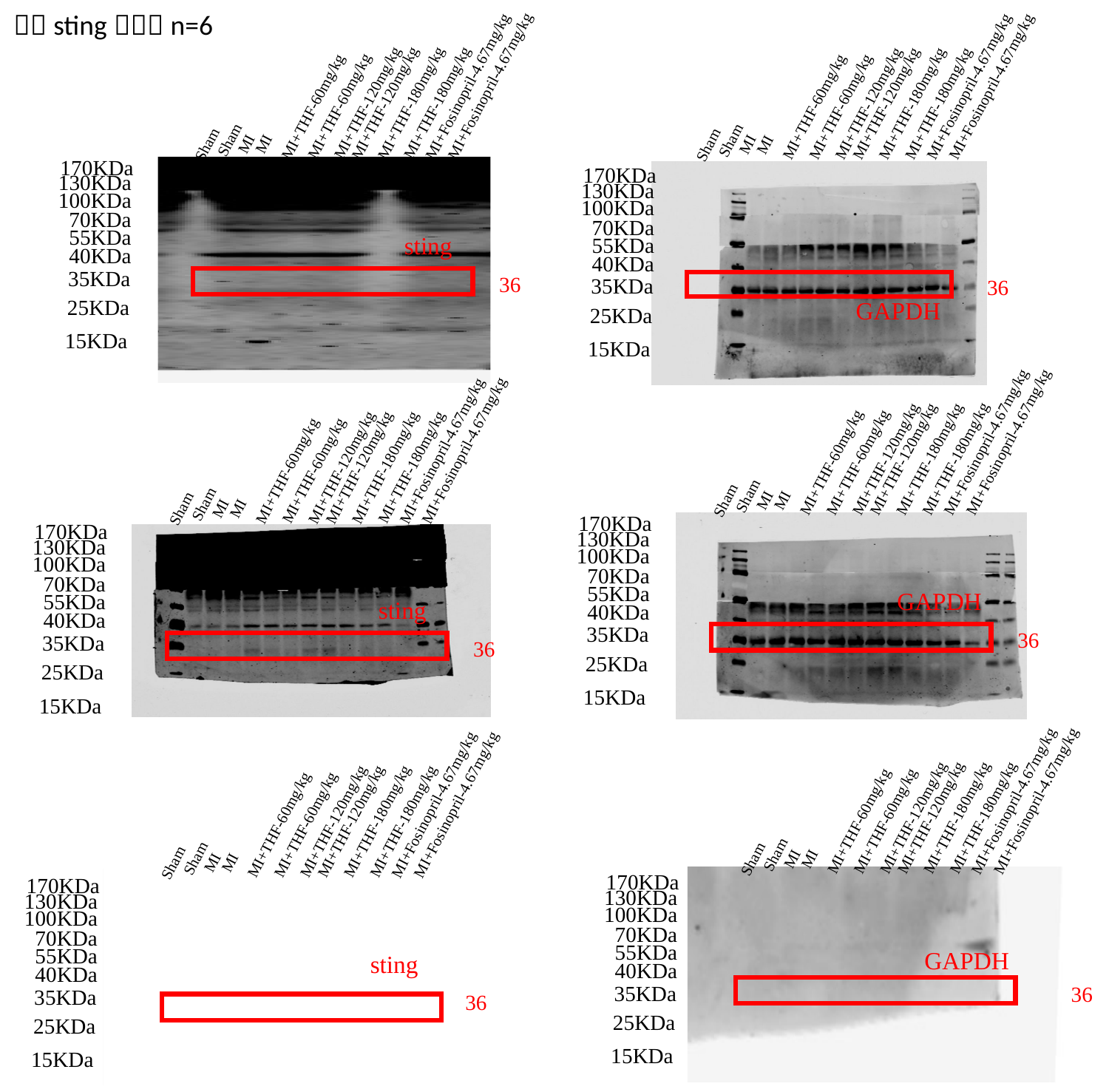

组织sting蛋白，n=6
MI+Fosinopril-4.67mg/kg
MI+Fosinopril-4.67mg/kg
MI+Fosinopril-4.67mg/kg
MI+Fosinopril-4.67mg/kg
MI+THF-60mg/kg
MI+THF-60mg/kg
MI+THF-180mg/kg
MI+THF-180mg/kg
MI+THF-120mg/kg
MI+THF-120mg/kg
MI+THF-180mg/kg
MI+THF-60mg/kg
MI+THF-60mg/kg
MI+THF-180mg/kg
MI+THF-120mg/kg
MI+THF-120mg/kg
Sham
Sham
MI
MI
MI
MI
Sham
Sham
170KDa
170KDa
130KDa
130KDa
100KDa
100KDa
70KDa
70KDa
55KDa
sting
55KDa
40KDa
40KDa
35KDa
36
35KDa
36
25KDa
GAPDH
25KDa
15KDa
15KDa
MI+Fosinopril-4.67mg/kg
MI+Fosinopril-4.67mg/kg
MI+THF-60mg/kg
MI+THF-180mg/kg
MI+THF-120mg/kg
MI+THF-180mg/kg
MI+THF-60mg/kg
MI+Fosinopril-4.67mg/kg
MI+Fosinopril-4.67mg/kg
MI+THF-60mg/kg
MI+THF-120mg/kg
MI+THF-180mg/kg
MI+THF-120mg/kg
MI+THF-60mg/kg
MI+THF-180mg/kg
MI+THF-120mg/kg
Sham
MI
MI
Sham
Sham
MI
MI
Sham
170KDa
170KDa
130KDa
130KDa
100KDa
100KDa
70KDa
70KDa
55KDa
GAPDH
55KDa
sting
40KDa
40KDa
35KDa
36
35KDa
36
25KDa
25KDa
15KDa
15KDa
MI+Fosinopril-4.67mg/kg
MI+Fosinopril-4.67mg/kg
MI+THF-60mg/kg
MI+THF-180mg/kg
MI+THF-120mg/kg
MI+Fosinopril-4.67mg/kg
MI+Fosinopril-4.67mg/kg
MI+THF-60mg/kg
MI+THF-60mg/kg
MI+THF-180mg/kg
MI+THF-180mg/kg
MI+THF-120mg/kg
MI+THF-60mg/kg
MI+THF-180mg/kg
MI+THF-120mg/kg
MI+THF-120mg/kg
Sham
Sham
MI
MI
MI
MI
Sham
Sham
170KDa
170KDa
130KDa
130KDa
100KDa
100KDa
70KDa
70KDa
55KDa
55KDa
GAPDH
sting
40KDa
40KDa
35KDa
36
35KDa
36
25KDa
25KDa
15KDa
15KDa

## Slide 3
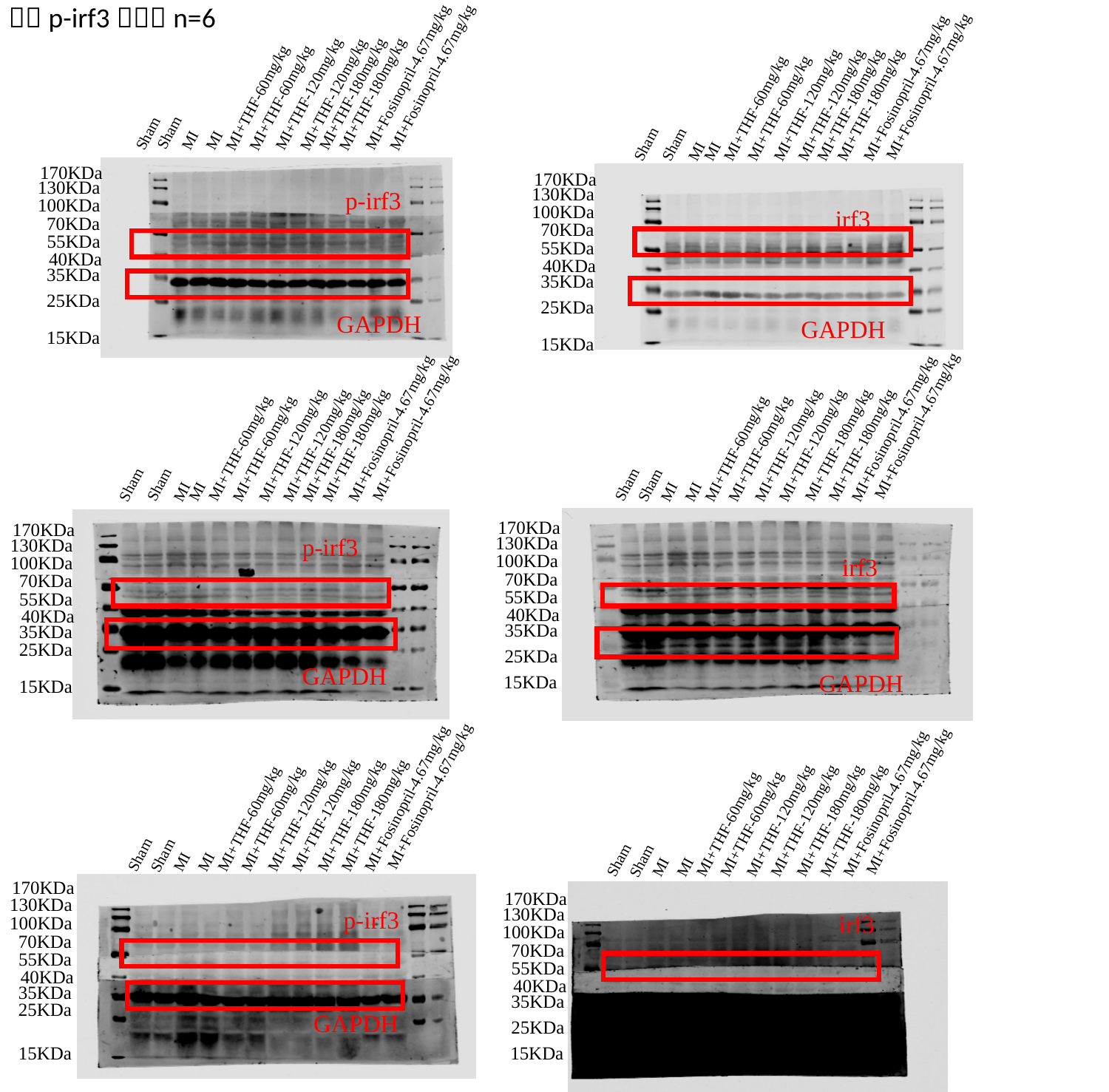

组织p-irf3蛋白，n=6
MI+Fosinopril-4.67mg/kg
MI+Fosinopril-4.67mg/kg
MI+THF-60mg/kg
MI+THF-180mg/kg
MI+THF-120mg/kg
MI+THF-60mg/kg
MI+THF-180mg/kg
MI+Fosinopril-4.67mg/kg
MI+THF-120mg/kg
MI+Fosinopril-4.67mg/kg
MI+THF-60mg/kg
MI+THF-180mg/kg
MI+THF-120mg/kg
MI+THF-60mg/kg
MI+THF-180mg/kg
MI+THF-120mg/kg
Sham
MI
Sham
MI
Sham
Sham
MI
MI
170KDa
170KDa
130KDa
130KDa
p-irf3
100KDa
100KDa
irf3
70KDa
70KDa
55KDa
55KDa
40KDa
40KDa
35KDa
35KDa
25KDa
25KDa
GAPDH
GAPDH
15KDa
15KDa
MI+Fosinopril-4.67mg/kg
MI+Fosinopril-4.67mg/kg
MI+Fosinopril-4.67mg/kg
MI+Fosinopril-4.67mg/kg
MI+THF-60mg/kg
MI+THF-60mg/kg
MI+THF-180mg/kg
MI+THF-180mg/kg
MI+THF-120mg/kg
MI+THF-120mg/kg
MI+THF-60mg/kg
MI+THF-60mg/kg
MI+THF-180mg/kg
MI+THF-180mg/kg
MI+THF-120mg/kg
MI+THF-120mg/kg
Sham
Sham
MI
Sham
MI
Sham
MI
MI
170KDa
170KDa
p-irf3
130KDa
130KDa
100KDa
100KDa
irf3
70KDa
70KDa
55KDa
55KDa
40KDa
40KDa
35KDa
35KDa
25KDa
25KDa
GAPDH
GAPDH
15KDa
15KDa
MI+Fosinopril-4.67mg/kg
MI+Fosinopril-4.67mg/kg
MI+THF-60mg/kg
MI+THF-180mg/kg
MI+Fosinopril-4.67mg/kg
MI+THF-120mg/kg
MI+Fosinopril-4.67mg/kg
MI+THF-60mg/kg
MI+THF-180mg/kg
MI+THF-60mg/kg
MI+THF-180mg/kg
MI+THF-120mg/kg
MI+THF-120mg/kg
MI+THF-60mg/kg
MI+THF-180mg/kg
MI+THF-120mg/kg
Sham
Sham
Sham
MI
MI
Sham
MI
MI
170KDa
170KDa
130KDa
130KDa
p-irf3
irf3
100KDa
100KDa
70KDa
70KDa
55KDa
55KDa
40KDa
40KDa
35KDa
35KDa
25KDa
GAPDH
25KDa
15KDa
15KDa

## Slide 4
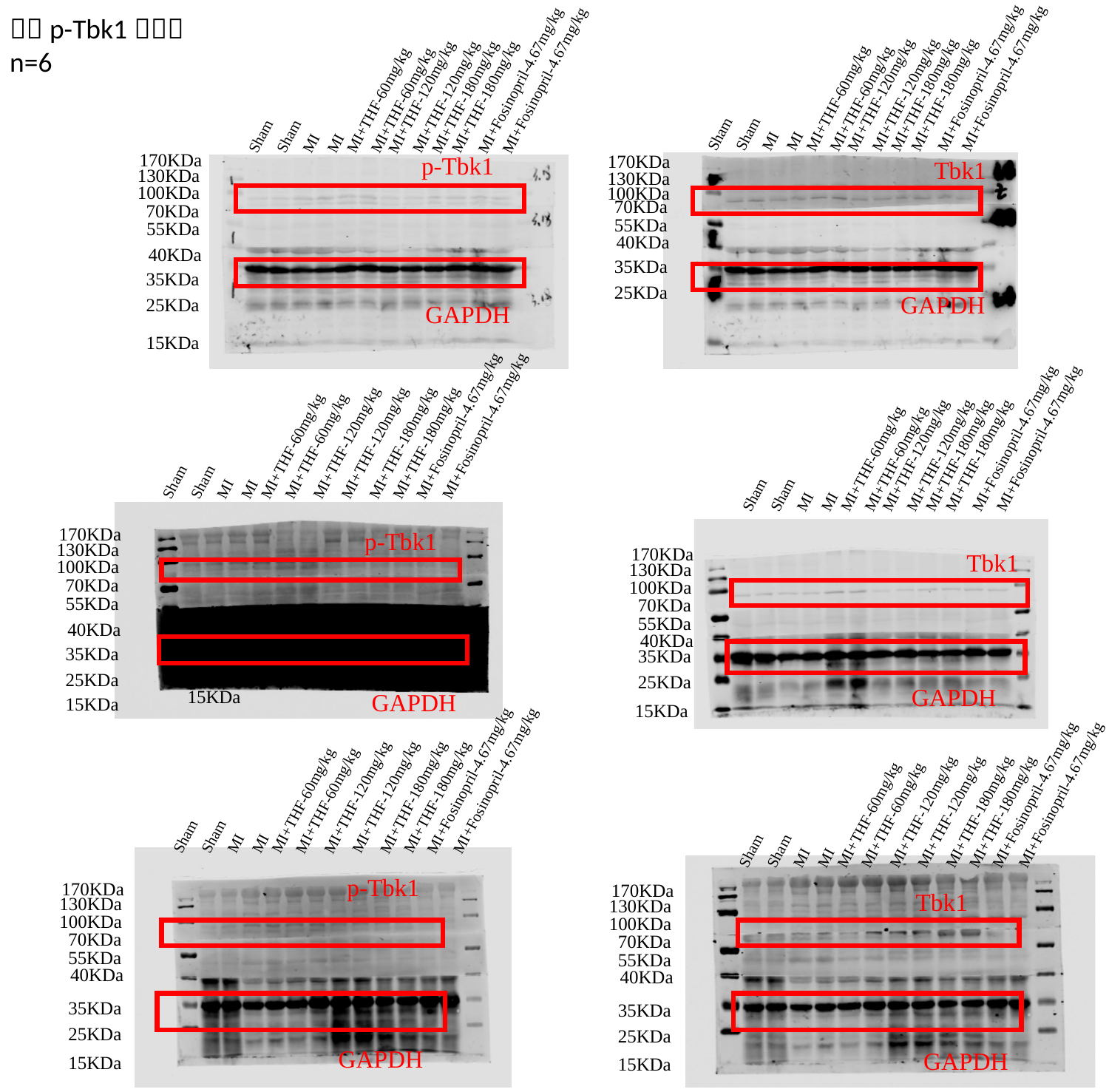

组织p-Tbk1蛋白，
n=6
MI+Fosinopril-4.67mg/kg
MI+Fosinopril-4.67mg/kg
MI+THF-60mg/kg
MI+Fosinopril-4.67mg/kg
MI+Fosinopril-4.67mg/kg
MI+THF-180mg/kg
MI+THF-60mg/kg
MI+THF-120mg/kg
MI+THF-60mg/kg
MI+THF-180mg/kg
MI+THF-180mg/kg
MI+THF-120mg/kg
MI+THF-60mg/kg
MI+THF-180mg/kg
MI+THF-120mg/kg
MI+THF-120mg/kg
Sham
Sham
MI
Sham
MI
Sham
MI
MI
170KDa
170KDa
p-Tbk1
Tbk1
130KDa
130KDa
100KDa
100KDa
70KDa
70KDa
55KDa
55KDa
40KDa
40KDa
35KDa
35KDa
25KDa
GAPDH
25KDa
GAPDH
15KDa
MI+Fosinopril-4.67mg/kg
MI+Fosinopril-4.67mg/kg
MI+THF-60mg/kg
MI+THF-180mg/kg
MI+THF-120mg/kg
MI+THF-60mg/kg
MI+THF-180mg/kg
MI+THF-120mg/kg
MI+Fosinopril-4.67mg/kg
MI+Fosinopril-4.67mg/kg
MI+THF-60mg/kg
MI+THF-180mg/kg
MI+THF-120mg/kg
MI+THF-60mg/kg
MI+THF-180mg/kg
MI+THF-120mg/kg
Sham
Sham
MI
MI
Sham
MI
Sham
MI
170KDa
p-Tbk1
130KDa
170KDa
Tbk1
100KDa
130KDa
70KDa
100KDa
55KDa
70KDa
55KDa
40KDa
40KDa
35KDa
35KDa
25KDa
25KDa
GAPDH
15KDa
GAPDH
15KDa
15KDa
MI+Fosinopril-4.67mg/kg
MI+Fosinopril-4.67mg/kg
MI+THF-60mg/kg
MI+THF-180mg/kg
MI+THF-120mg/kg
MI+THF-60mg/kg
MI+THF-180mg/kg
MI+THF-120mg/kg
MI+Fosinopril-4.67mg/kg
MI+Fosinopril-4.67mg/kg
MI+THF-60mg/kg
MI+THF-180mg/kg
MI+THF-120mg/kg
MI+THF-60mg/kg
MI+THF-180mg/kg
MI+THF-120mg/kg
Sham
Sham
MI
MI
Sham
Sham
MI
MI
p-Tbk1
170KDa
170KDa
Tbk1
130KDa
130KDa
100KDa
100KDa
70KDa
70KDa
55KDa
55KDa
40KDa
40KDa
35KDa
35KDa
25KDa
25KDa
GAPDH
GAPDH
15KDa
15KDa
