# Supplementary material for: Tianhuang formula attenuates cardiomyocyte pyroptosis in myocardial infarction by suppressing oxidative stress and the cGAS–STING–NLRP3 axis
Source: Front Immunol. 2026 Feb 20;17:1761299. doi: 10.3389/fimmu.2026.1761299 (PMC12965622; doi:10.3389/fimmu.2026.1761299)
Supplement: Supplementary file 4 [file DataSheet4.zip › WB-Raw data/Figure 6A WB.pptx]

## Slide 1
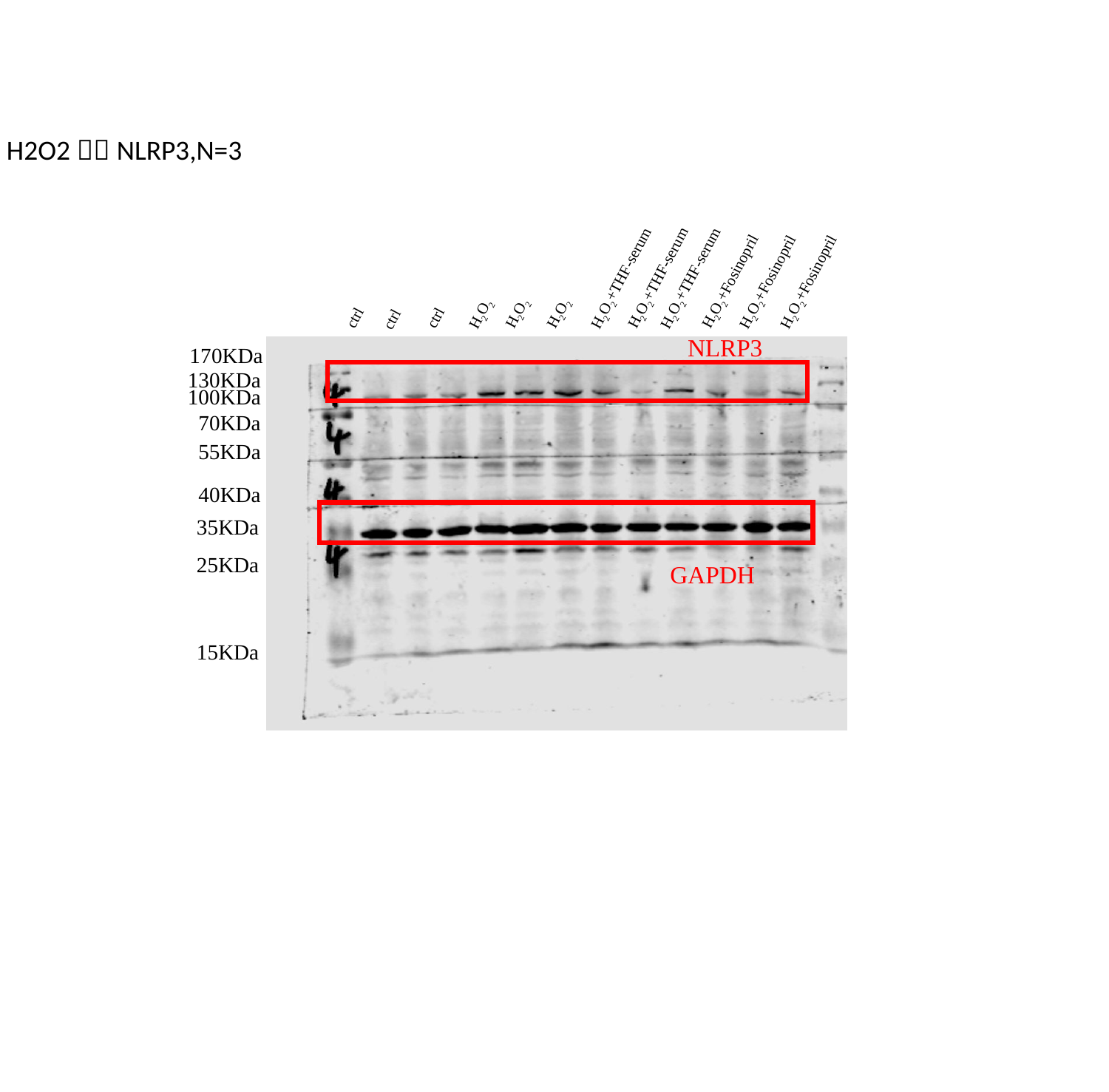

H2O2细胞NLRP3,N=3
H2O2+THF-serum
H2O2+Fosinopril
H2O2+Fosinopril
H2O2+THF-serum
H2O2+Fosinopril
H2O2+THF-serum
H2O2
H2O2
ctrl
ctrl
ctrl
H2O2
NLRP3
170KDa
130KDa
100KDa
70KDa
55KDa
40KDa
35KDa
25KDa
GAPDH
15KDa

## Slide 2
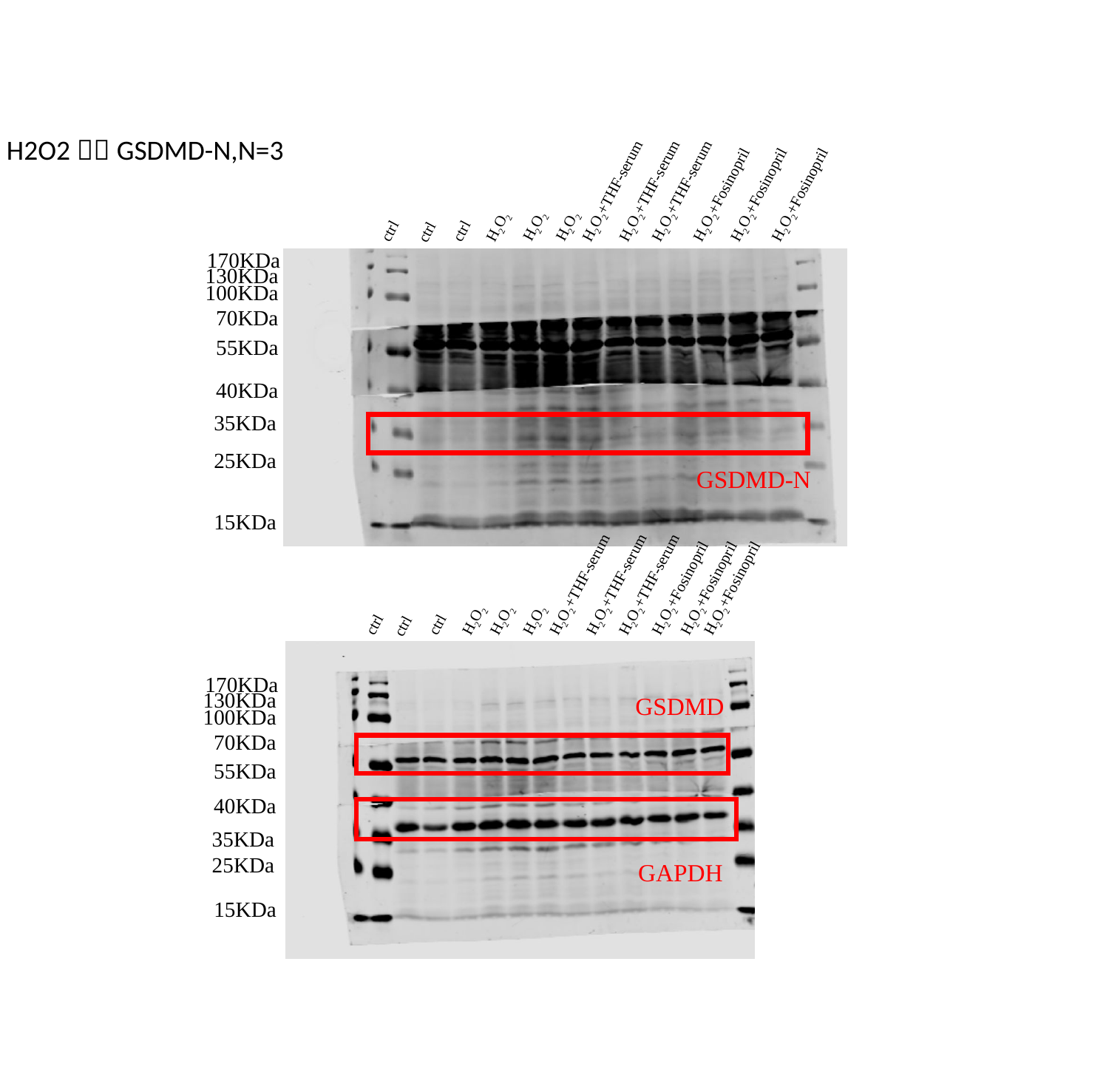

H2O2细胞GSDMD-N,N=3
H2O2+THF-serum
H2O2+Fosinopril
H2O2+THF-serum
H2O2+Fosinopril
H2O2+Fosinopril
H2O2+THF-serum
H2O2
H2O2
ctrl
ctrl
ctrl
H2O2
170KDa
130KDa
100KDa
70KDa
55KDa
40KDa
35KDa
25KDa
GSDMD-N
15KDa
H2O2+THF-serum
H2O2+Fosinopril
H2O2+THF-serum
H2O2+Fosinopril
H2O2+Fosinopril
H2O2+THF-serum
H2O2
H2O2
ctrl
ctrl
ctrl
H2O2
170KDa
130KDa
GSDMD
100KDa
70KDa
55KDa
40KDa
35KDa
25KDa
GAPDH
15KDa

## Slide 3
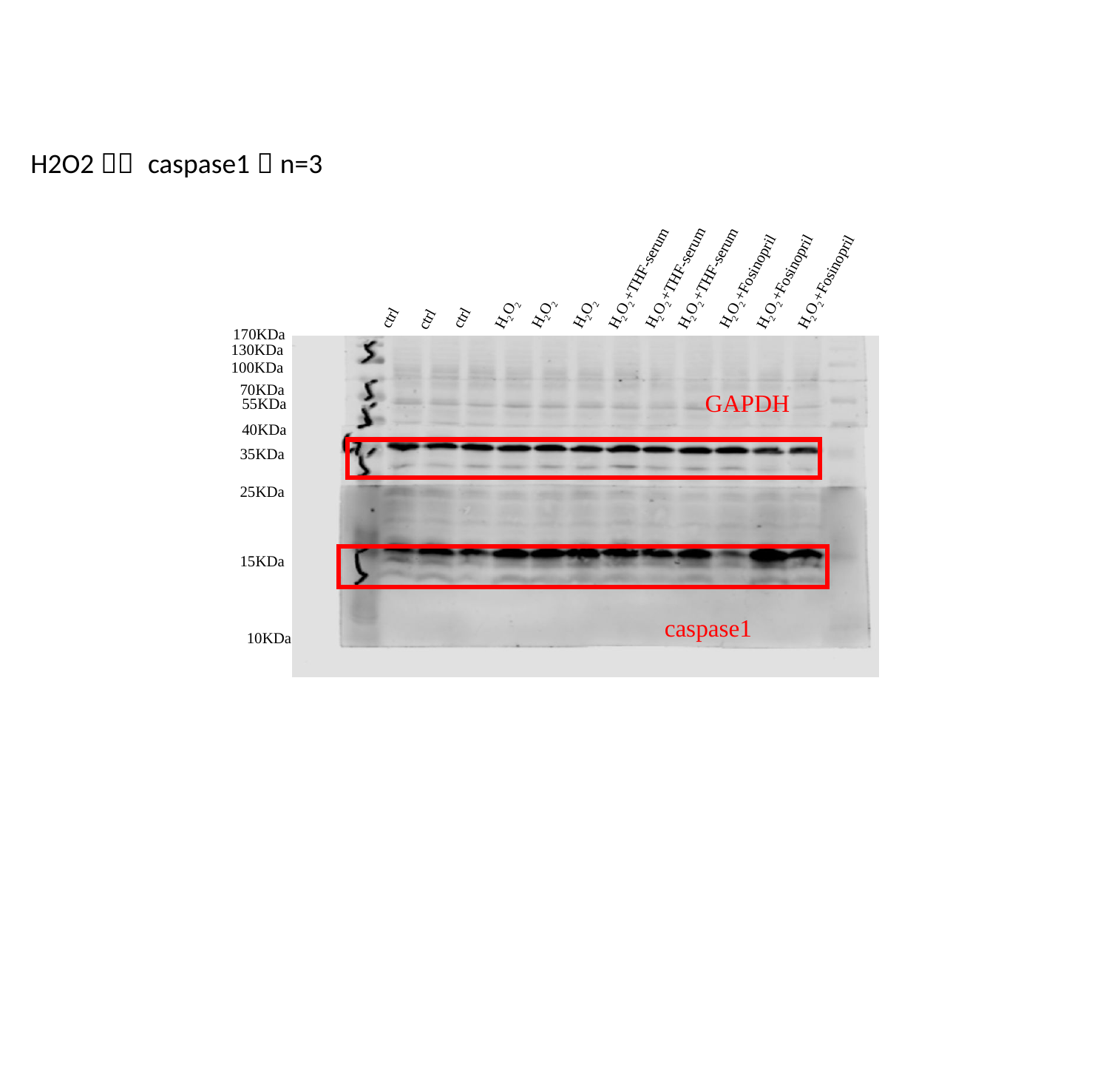

H2O2细胞 caspase1，n=3
H2O2+THF-serum
H2O2+Fosinopril
H2O2+THF-serum
H2O2+Fosinopril
H2O2+Fosinopril
H2O2+THF-serum
H2O2
H2O2
ctrl
ctrl
ctrl
H2O2
170KDa
130KDa
100KDa
70KDa
GAPDH
55KDa
40KDa
35KDa
25KDa
15KDa
caspase1
10KDa
