# Supplementary material for: Tianhuang formula attenuates cardiomyocyte pyroptosis in myocardial infarction by suppressing oxidative stress and the cGAS–STING–NLRP3 axis
Source: Front Immunol. 2026 Feb 20;17:1761299. doi: 10.3389/fimmu.2026.1761299 (PMC12965622; doi:10.3389/fimmu.2026.1761299)
Supplement: Supplementary file 4 [file DataSheet4.zip › WB-Raw data/Figure 4N WB.pptx]

## Slide 1
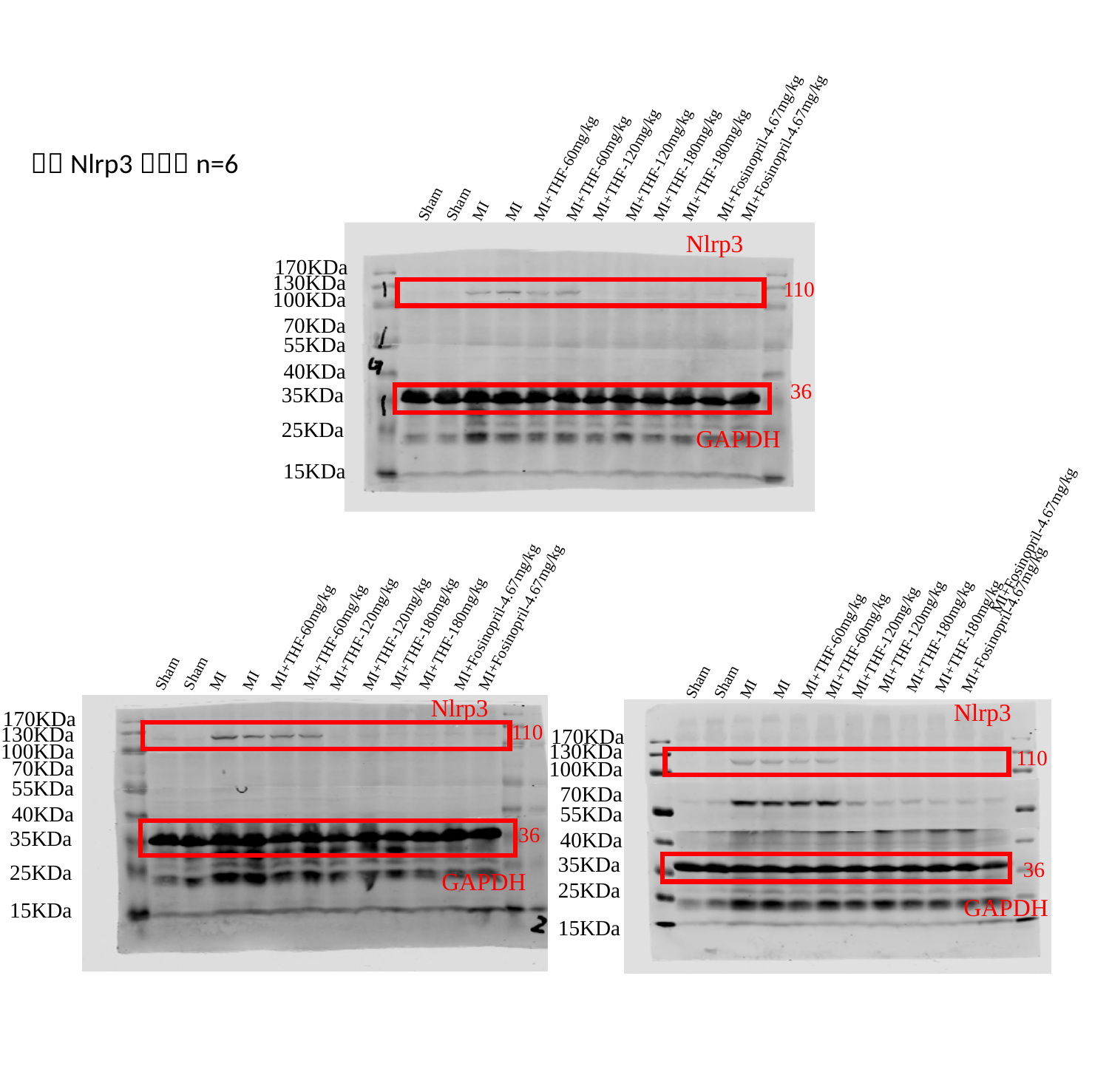

MI+Fosinopril-4.67mg/kg
MI+Fosinopril-4.67mg/kg
MI+THF-60mg/kg
MI+THF-180mg/kg
MI+THF-120mg/kg
MI+THF-60mg/kg
MI+THF-180mg/kg
MI+THF-120mg/kg
组织Nlrp3蛋白，n=6
Sham
Sham
MI
MI
Nlrp3
170KDa
130KDa
110
100KDa
70KDa
55KDa
40KDa
36
35KDa
25KDa
GAPDH
15KDa
MI+Fosinopril-4.67mg/kg
MI+Fosinopril-4.67mg/kg
MI+Fosinopril-4.67mg/kg
MI+THF-60mg/kg
MI+Fosinopril-4.67mg/kg
MI+THF-180mg/kg
MI+THF-120mg/kg
MI+THF-180mg/kg
MI+THF-60mg/kg
MI+THF-180mg/kg
MI+THF-120mg/kg
MI+THF-180mg/kg
MI+THF-60mg/kg
MI+THF-120mg/kg
MI+THF-60mg/kg
MI+THF-120mg/kg
Sham
MI
Sham
MI
Sham
Sham
MI
MI
Nlrp3
Nlrp3
170KDa
110
130KDa
170KDa
100KDa
130KDa
110
70KDa
100KDa
55KDa
70KDa
40KDa
55KDa
36
35KDa
40KDa
35KDa
36
25KDa
GAPDH
25KDa
GAPDH
15KDa
15KDa

## Slide 2
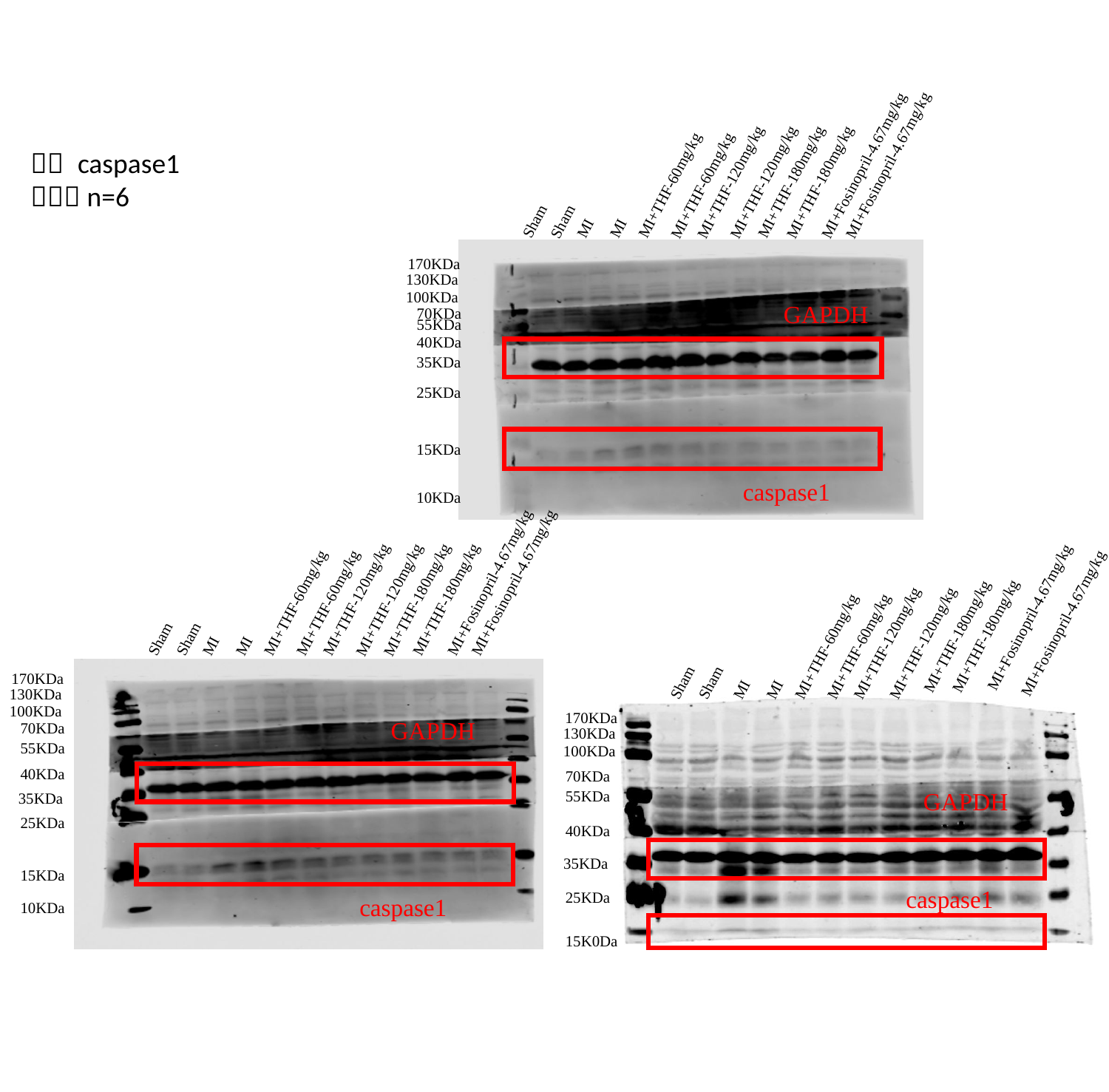

组织 caspase1
蛋白，n=6
MI+Fosinopril-4.67mg/kg
MI+Fosinopril-4.67mg/kg
MI+THF-60mg/kg
MI+THF-180mg/kg
MI+THF-120mg/kg
MI+THF-60mg/kg
MI+THF-180mg/kg
MI+THF-120mg/kg
Sham
MI
Sham
MI
170KDa
130KDa
100KDa
GAPDH
70KDa
55KDa
40KDa
35KDa
25KDa
15KDa
caspase1
10KDa
MI+Fosinopril-4.67mg/kg
MI+Fosinopril-4.67mg/kg
MI+THF-60mg/kg
MI+THF-180mg/kg
MI+THF-120mg/kg
MI+THF-60mg/kg
MI+THF-180mg/kg
MI+THF-120mg/kg
MI+Fosinopril-4.67mg/kg
MI+THF-180mg/kg
MI+Fosinopril-4.67mg/kg
MI+THF-180mg/kg
MI+THF-60mg/kg
MI+THF-120mg/kg
MI+THF-60mg/kg
Sham
MI+THF-120mg/kg
Sham
MI
MI
Sham
Sham
MI
MI
170KDa
130KDa
100KDa
170KDa
GAPDH
70KDa
130KDa
55KDa
100KDa
40KDa
70KDa
55KDa
GAPDH
35KDa
25KDa
40KDa
35KDa
15KDa
caspase1
25KDa
caspase1
10KDa
15K0Da

## Slide 3
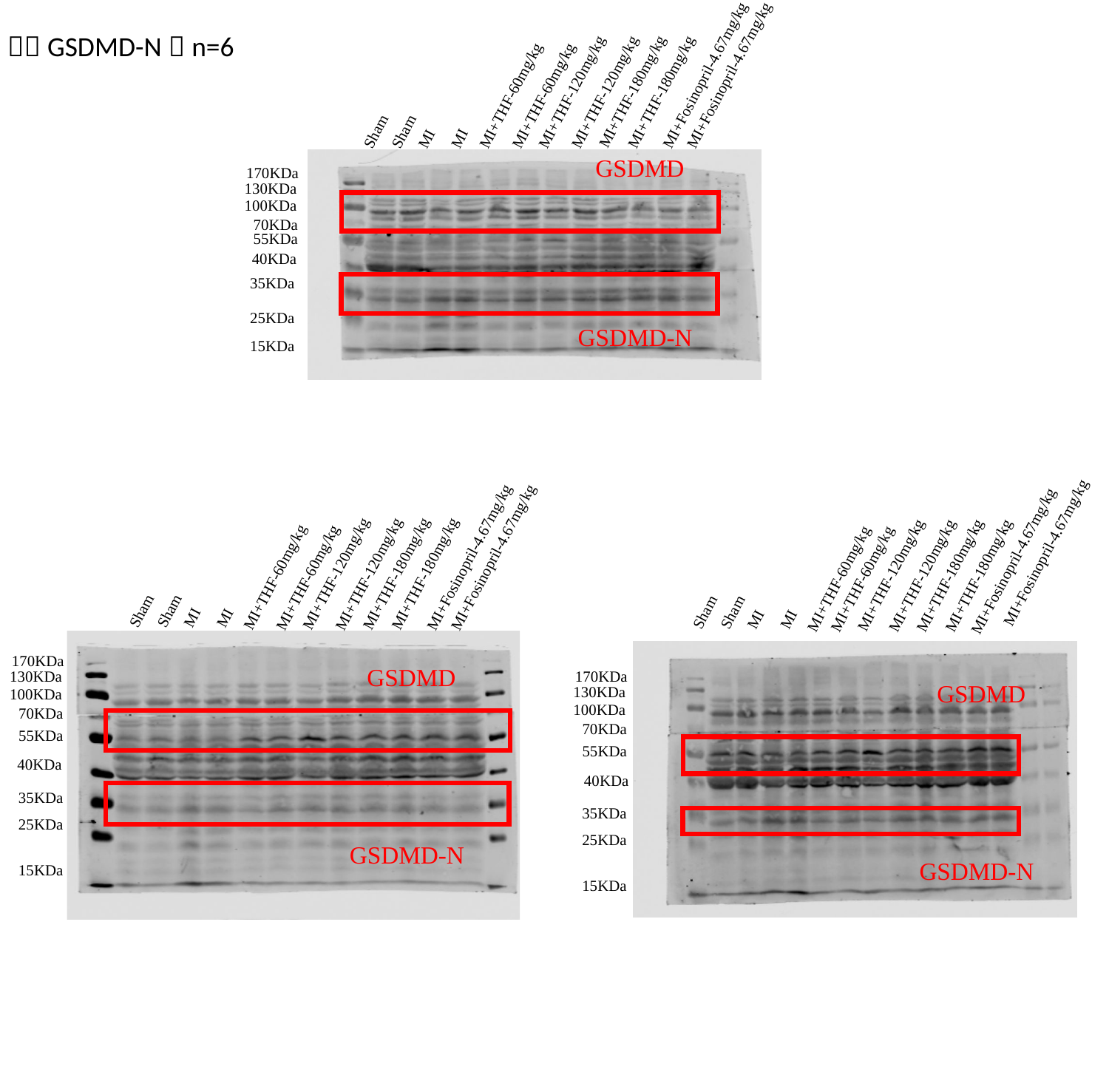

组织GSDMD-N，n=6
MI+Fosinopril-4.67mg/kg
MI+Fosinopril-4.67mg/kg
MI+THF-60mg/kg
MI+THF-180mg/kg
MI+THF-120mg/kg
MI+THF-60mg/kg
MI+THF-180mg/kg
MI+THF-120mg/kg
Sham
MI
Sham
MI
GSDMD
170KDa
130KDa
100KDa
70KDa
55KDa
40KDa
35KDa
25KDa
GSDMD-N
15KDa
MI+Fosinopril-4.67mg/kg
MI+Fosinopril-4.67mg/kg
MI+Fosinopril-4.67mg/kg
MI+THF-60mg/kg
MI+THF-60mg/kg
MI+THF-180mg/kg
MI+THF-120mg/kg
MI+Fosinopril-4.67mg/kg
MI+THF-180mg/kg
MI+THF-60mg/kg
MI+THF-120mg/kg
MI+THF-180mg/kg
MI+THF-60mg/kg
MI+THF-180mg/kg
MI+THF-120mg/kg
MI+THF-120mg/kg
Sham
Sham
MI
MI
Sham
Sham
MI
MI
170KDa
GSDMD
130KDa
170KDa
GSDMD
130KDa
100KDa
100KDa
70KDa
70KDa
55KDa
55KDa
40KDa
40KDa
35KDa
35KDa
25KDa
25KDa
GSDMD-N
GSDMD-N
15KDa
15KDa

## Slide 4
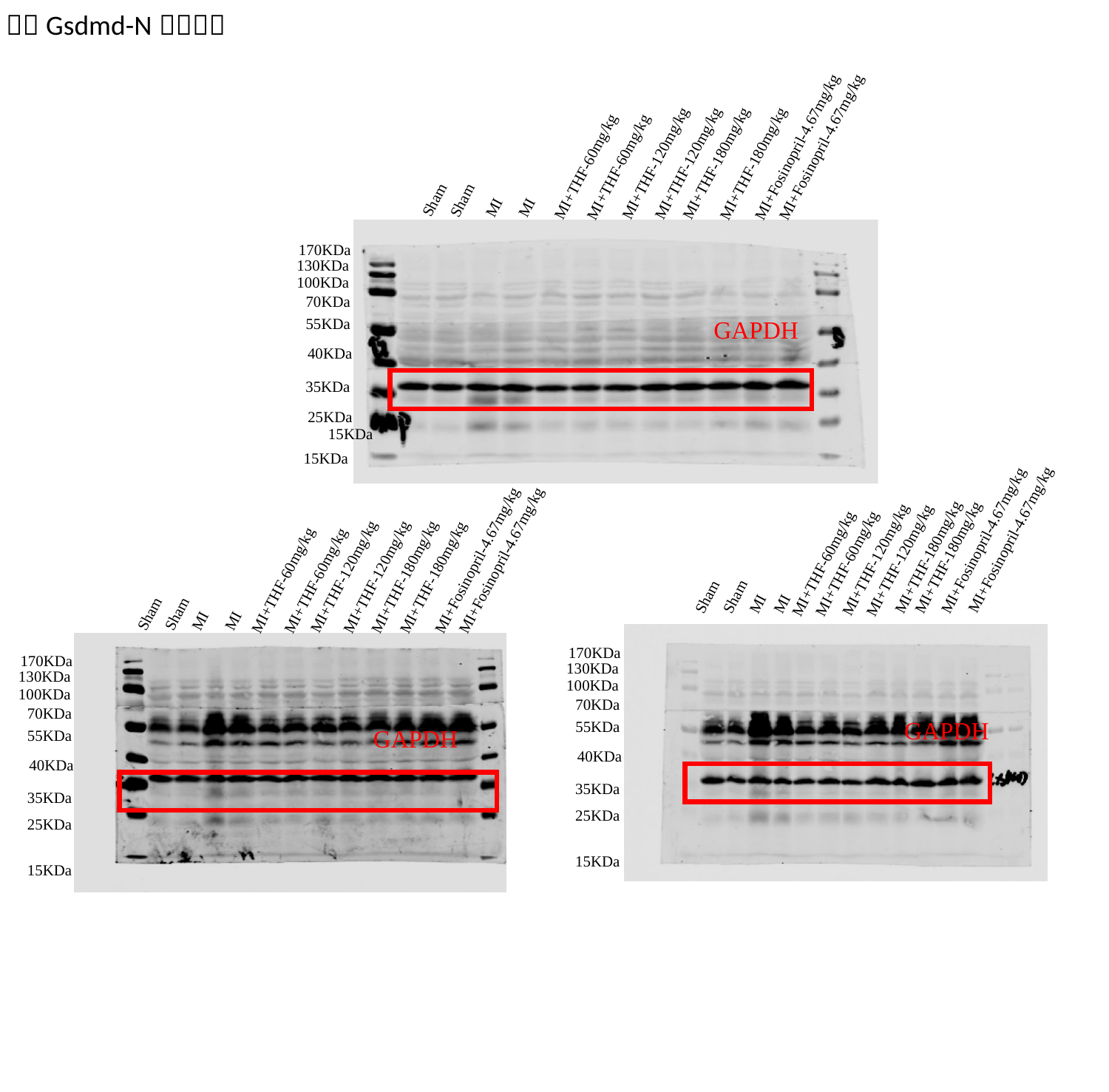

组织Gsdmd-N对应内参
MI+Fosinopril-4.67mg/kg
MI+Fosinopril-4.67mg/kg
MI+THF-60mg/kg
MI+THF-180mg/kg
MI+THF-120mg/kg
MI+THF-60mg/kg
MI+THF-180mg/kg
MI+THF-120mg/kg
Sham
Sham
MI
MI
170KDa
130KDa
100KDa
70KDa
55KDa
GAPDH
40KDa
35KDa
25KDa
15KDa
15KDa
MI+Fosinopril-4.67mg/kg
MI+Fosinopril-4.67mg/kg
MI+THF-180mg/kg
MI+THF-60mg/kg
MI+THF-180mg/kg
MI+THF-120mg/kg
MI+THF-60mg/kg
MI+THF-120mg/kg
MI+Fosinopril-4.67mg/kg
MI+Fosinopril-4.67mg/kg
MI+THF-60mg/kg
MI+THF-180mg/kg
MI+THF-120mg/kg
MI+THF-60mg/kg
MI+THF-180mg/kg
MI+THF-120mg/kg
Sham
MI
MI
Sham
Sham
Sham
MI
MI
170KDa
170KDa
130KDa
130KDa
100KDa
100KDa
70KDa
70KDa
GAPDH
55KDa
GAPDH
55KDa
40KDa
40KDa
35KDa
35KDa
25KDa
25KDa
15KDa
15KDa
