# Supplementary material for: Tianhuang formula attenuates cardiomyocyte pyroptosis in myocardial infarction by suppressing oxidative stress and the cGAS–STING–NLRP3 axis
Source: Front Immunol. 2026 Feb 20;17:1761299. doi: 10.3389/fimmu.2026.1761299 (PMC12965622; doi:10.3389/fimmu.2026.1761299)
Supplement: Supplementary file 11 [file Supplementaryfile1.docx]

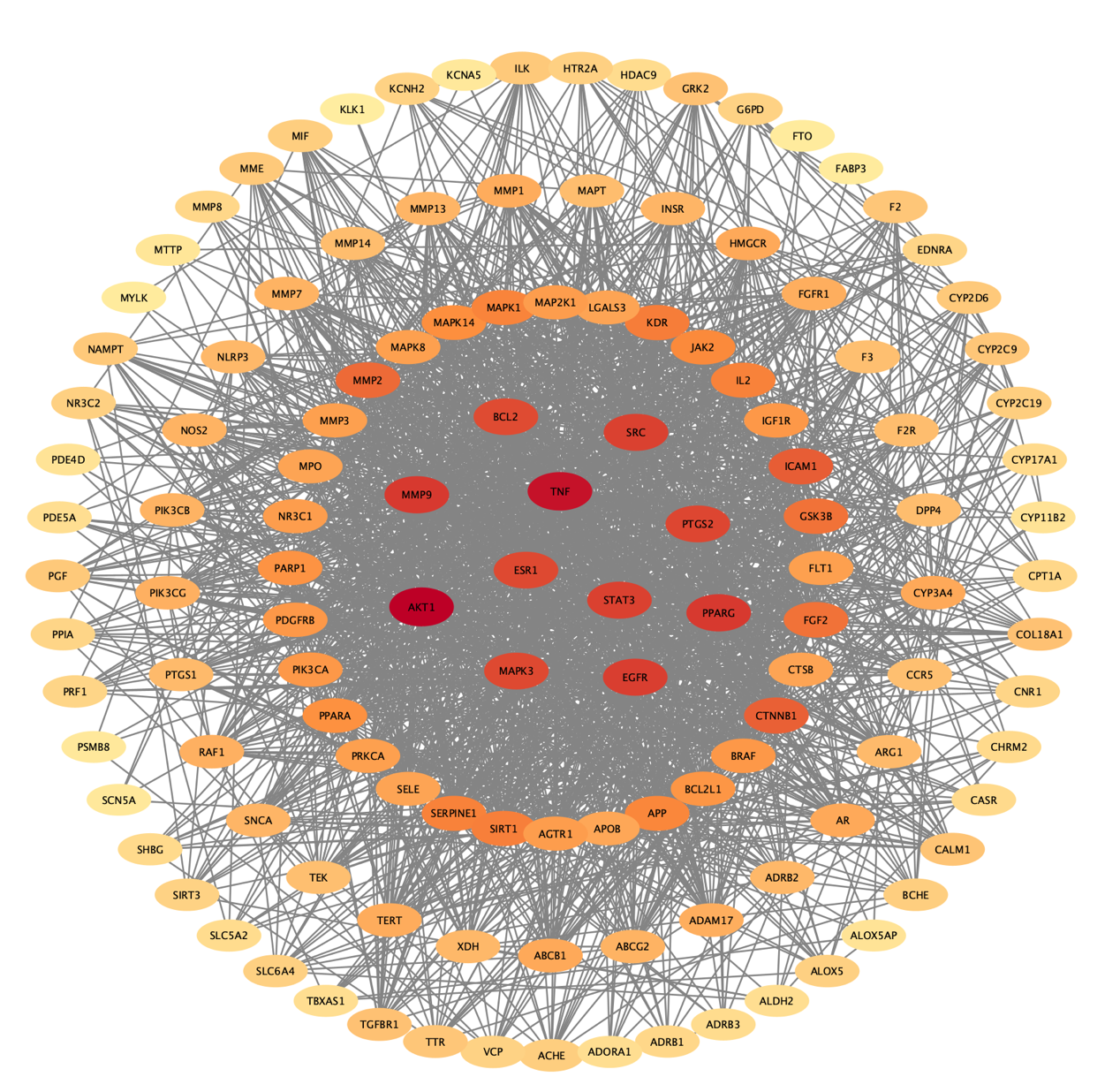


Figure S1. PPI network of THF-related targets in MI. The network highlights key targets, including AKT1, TNF, STAT3, and PTGS2, which may play central roles in the regulatory effects of THF on MI.


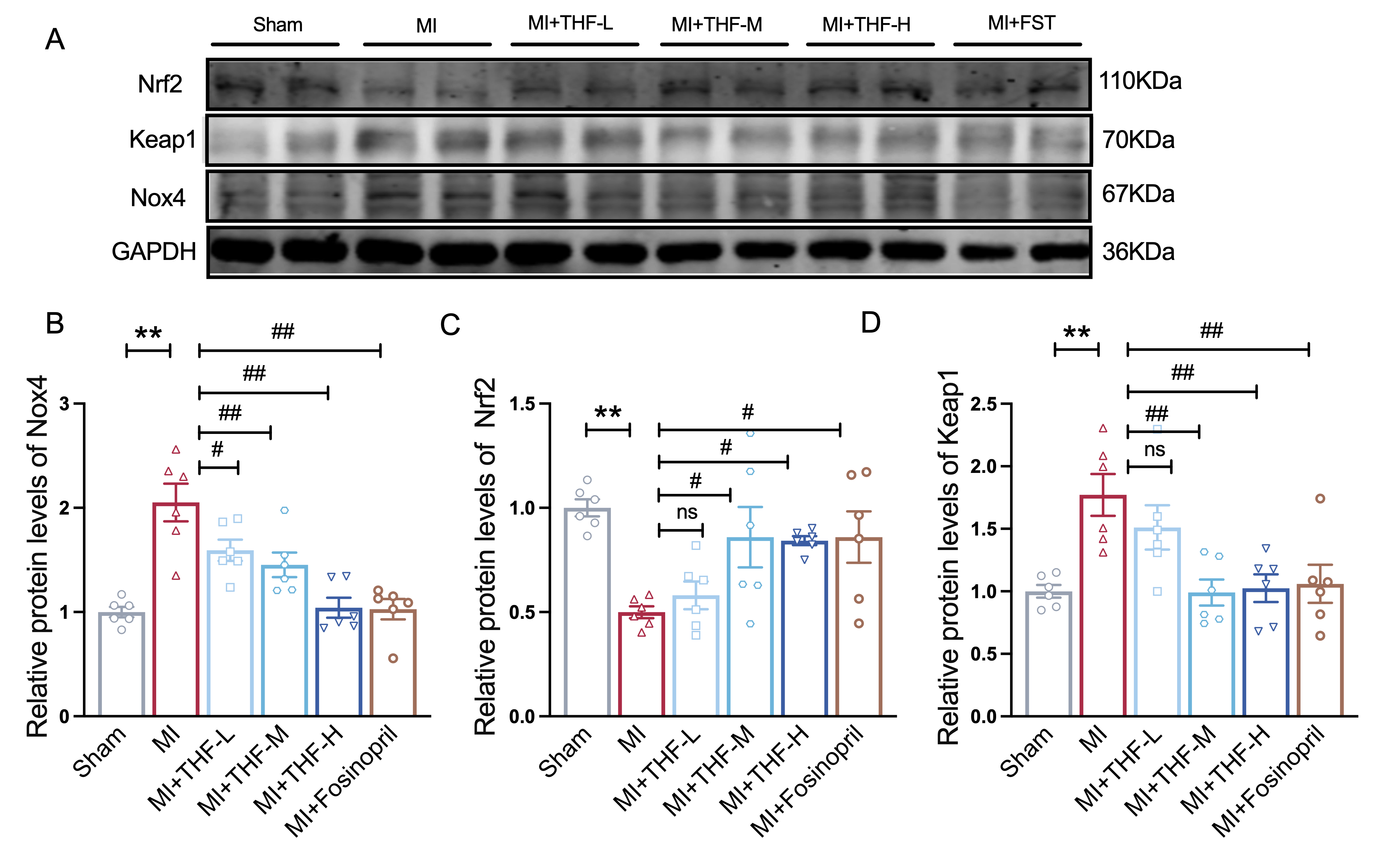


Figure S2. THF alleviates oxidative stress in the cardiac tissue of MI mice by inhibiting the Nox4/Keap1/Nrf2 signaling pathway. (A-D) Western blotting was employed to determine the protein expression of Nox4, Nrf2 and Keap1 within cardiac tissue. GAPDH was utilized as a loading control for normalization (n = 6 per group). Data are presented as mean ± SEM. ^*^*P* < 0.05 vs. Sham group, ^**^*P* < 0.01 vs. Sham group; ^#^*P* < 0.05 vs. MI group, ^##^*P* < 0.01 vs. MI group.


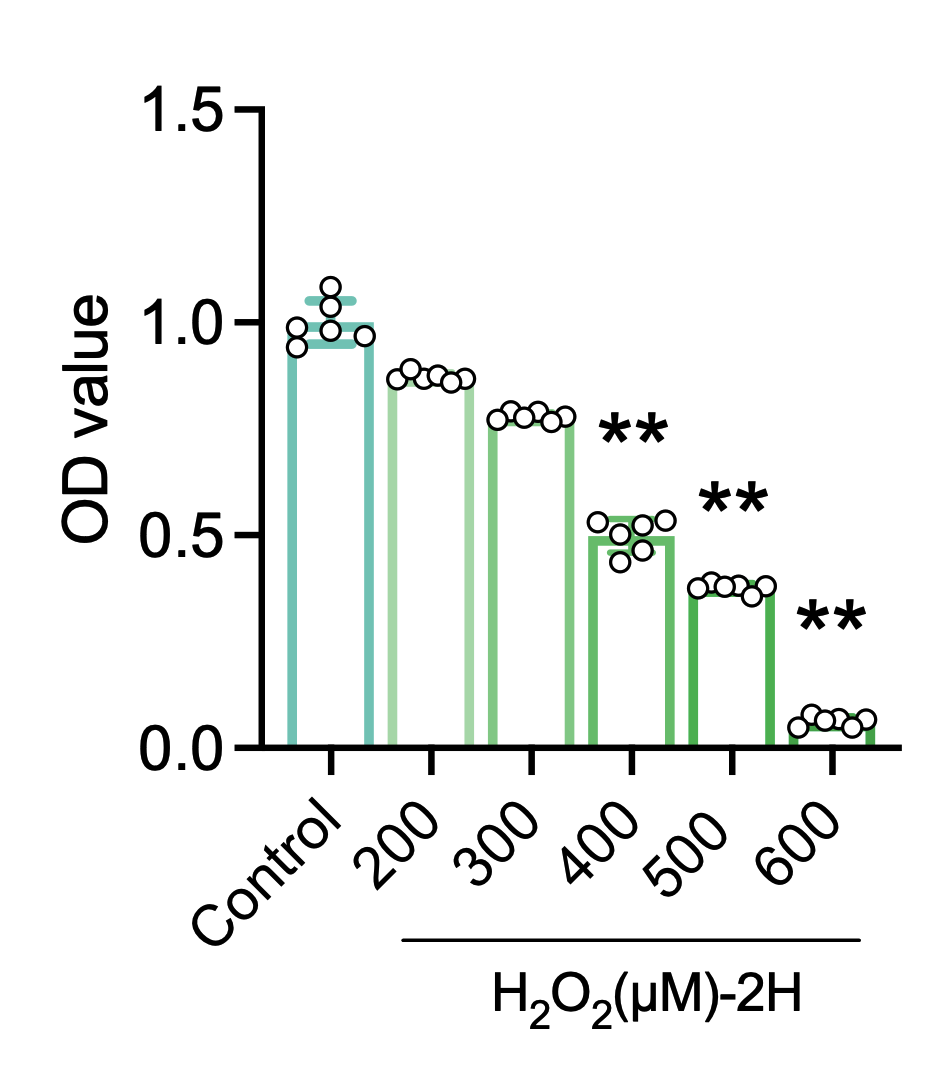


Figure. S3. CCK-8 assay evaluating the effect of THF concentration on cell viability (n=8 per group). Data are presented as mean ± SEM. ^*^*P* < 0.05 vs. the control group; ^**^*P* < 0.01 vs. the control group; ^#^*P* < 0.05 vs. the H₂O₂ group; ^##^*P* < 0.01 vs. the H₂O₂ group.


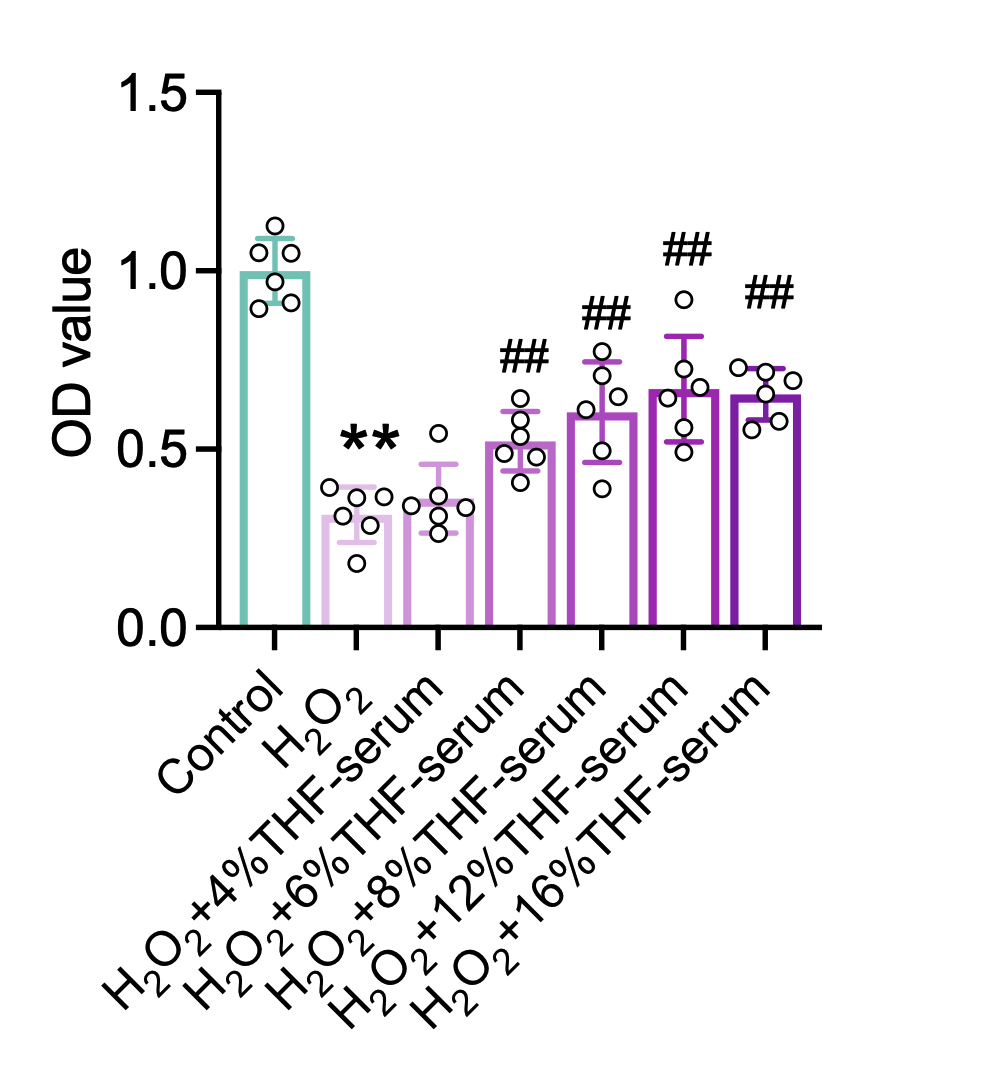


Figure. S4. CCK-8 assay assessing the effect of H₂O₂ concentration on cell viability. Data are presented as mean ± SEM. ^*^*P* < 0.05 vs. the control group; ^**^*P* < 0.01 vs. the control group; ^#^*P* < 0.05 vs. the H₂O₂ group; ^##^*P* < 0.01 vs. the H₂O₂ group.


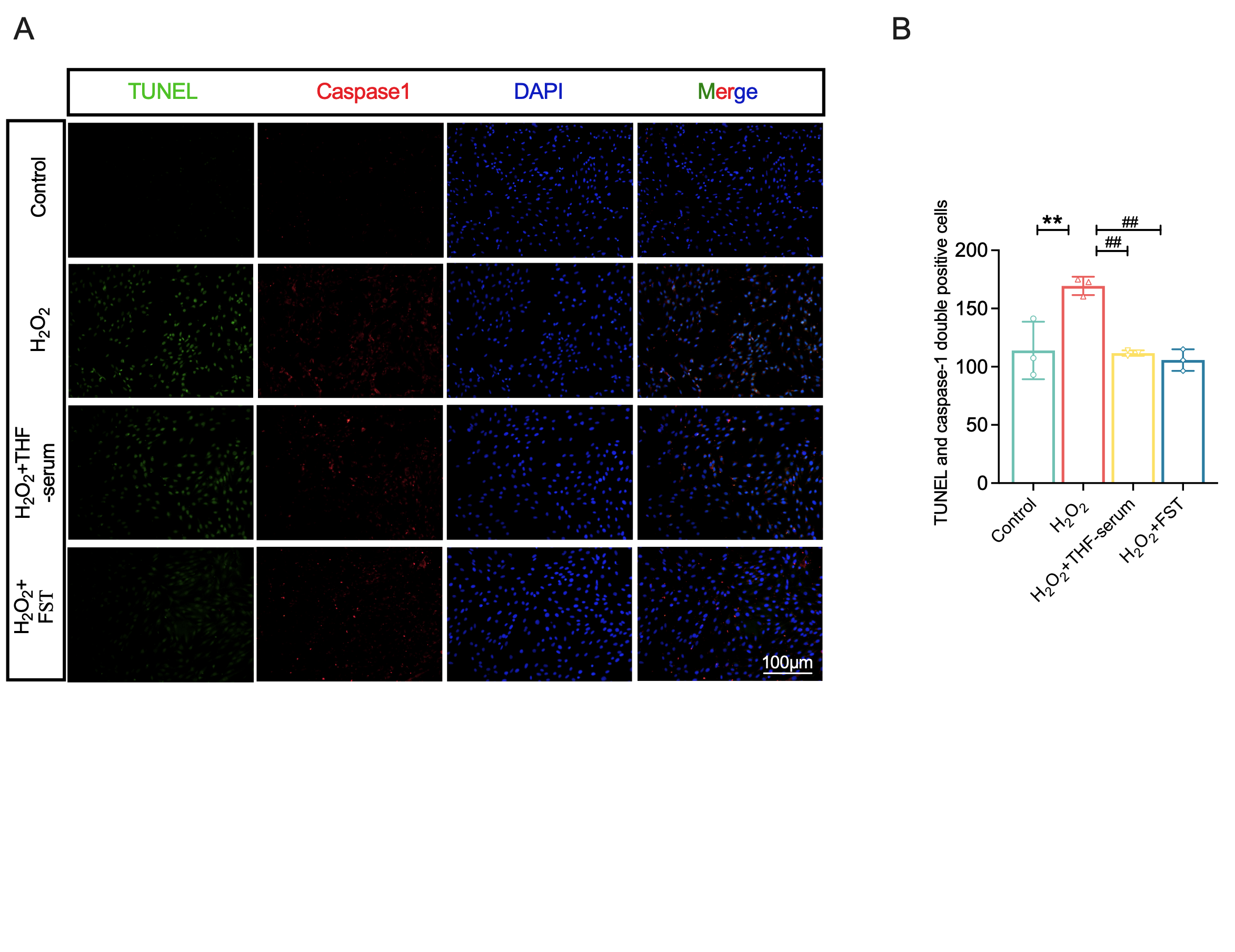


Figure. S5. (A–B) Representative images and quantification of caspase-1 (red) and TUNEL (green) double-positive cells in H₂O₂-treated H9c2 cells (n=3 per group). Data are presented as mean ± SEM. ^*^*P* < 0.05 vs. the control group; ^**^*P* < 0.01 vs. the control group; ^#^*P* < 0.05 vs. the H₂O₂ group; ^##^*P* < 0.01 vs. the H₂O₂ group.

| **Table S1 Primers for qRT-PCR**   \| Species \| Gene \| Forward \| Reverse \| \| --- \| --- \| --- \| --- \| \|  \| IL-1β \| GCCTCGTGCTGTCGGACCCATAT \| TCCTTTGAGGCCCAAGGCCACA \| \|  \| IL-18 \| GACTCTTGCGTCAACTTCAAGG \| CAGGCTGTCTTTTGTCAACGA \| \|  \| TNF-α \| CCCTCCTGGCCAACGGCATG \| TCGGGGCAGCCTTGTCCCTT \| \|  \| NLRP3 \| CTCGCATTGGTTCTGAGCTC \| TCGCAGCAAAGATCCACACAG \| \|  \| ANP \| AAGAACCTGCTAGACCACCTGGAG \| TGCTTCCTCAGTCTGCTCACTCAG \| \|  \| BNP \| GGAAGTCCTAGCCAGTCTCCAGAG \| GCCTTGGTCCTTCAAGAGCTGTC \| \|  \| β-MHC \| GCAAGACGGTGACTGTGAAGGAG \| GGTTGACGGTGACGCAGAAGAG \| \| Mouse \| SOD-1 \| CCATCAGTATGGGGACAATACA \| GGTCTCCAACATGCCTCTCT \| \|  \| HO-1 \| GGAGAATGGCAAGAATGAAGA \| CCGCAGGAAGGTAAAGAG \| \|  \| GPX-1 \| GCAGAGGTCCAGAAGAATGG \| AGCATCCACCCAAATGACAC \| \|  \| GAPDH \| AACTTTGGCATTGTGGAAGG \| CACATTGGGGGTAGGAACAC \| \|  \| NLRP3 \| CTCGCATTGGTTCTGAGCTC \| AGTAAGGCCGGAATTCACCA \| \| Rat \| IL-18 \| ACGGAGCATAAATGACCAAGTTC \| TCTGGGATTCGTTGGCTGTT \| \|  \| IL-1β \| GGGATGATGACGACCTGCTA \| TGTCGTTGCTTGTCTCTCCT \| \|  \| TNF-α \| ACACACGAGACGCTGAAGTA \| GGAACAGTCTGGGAAGCTCT \| \|  \| ANP \| GGGAAGTCAACCCGTCTCA \| GGCTCCAATCCTGTCAATCC \| \|  \| BNP \| CTCCAGAACAATCCACGATG \| ACAGCCCAAGCGACTGACT \| \|  \| β-MHC \| GCTGCTGGAGCTGATAAGAGAA \| GTTCTTTTGTAGGGCCTTGGTC \| \|  \| GAPDH \| CCAAGGTCATCCATGACAACTT \| AGGGGCCATCCACAGTCTT \|   **Table S2.The ID,OB and DL ofcompoundsin THF.** | | | | | | |
| --- | --- | --- | --- | --- | --- | --- | --- | --- | --- | --- | --- | --- | --- | --- | --- | --- | --- | --- | --- | --- | --- | --- | --- | --- | --- | --- | --- | --- | --- | --- | --- | --- | --- | --- | --- | --- | --- | --- | --- | --- | --- | --- | --- | --- | --- | --- | --- | --- | --- | --- | --- | --- | --- | --- | --- | --- | --- | --- | --- | --- | --- | --- | --- | --- | --- | --- | --- | --- | --- | --- | --- | --- | --- | --- | --- | --- | --- | --- | --- | --- | --- | --- | --- | --- | --- | --- |
| MolID | MoleculeName | MV | AlogP | OB(%) | DL | TCM |
| MOL000358 | [beta-sitosterol](https://old.tcmsp-e.com/molecule.php?qn=358) | 414.79 | 8.08 | 36.91 | 0.75 | SQ1 |
| MOL001792 | [DFV](https://old.tcmsp-e.com/molecule.php?qn=1792) | 256.27 | 2.57 | 32.76 | 0.18 | SQ2 |
| MOL002879 | [Diop](https://old.tcmsp-e.com/molecule.php?qn=2879) | 390.62 | 7.44 | 43.59 | 0.39 | SQ3 |
| MOL007475 | [ginsenosidef2](https://old.tcmsp-e.com/molecule.php?qn=7475) | 785.14 | 2.3 | 36.43 | 0.25 | SQ4 |
| MOL005344 | [ginsenosiderh2](https://old.tcmsp-e.com/molecule.php?qn=5344) | 622.98 | 4.04 | 36.32 | 0.56 | SQ5 |
| MOL001494 | [Mandenol](https://old.tcmsp-e.com/molecule.php?qn=1494) | 308.56 | 6.99 | 42 | 0.19 | SQ6 |
| MOL000098 | [quercetin](https://old.tcmsp-e.com/molecule.php?qn=98) | 302.25 | 1.5 | 46.43 | 0.28 | SQ7（HL13） |
| MOL000449 | [Stigmasterol](https://old.tcmsp-e.com/molecule.php?qn=449) | 412.77 | 7.64 | 43.83 | 0.76 | SQ8 |
| MOL002903 | [(R)-Canadine](https://old.tcmsp-e.com/molecule.php?qn=2903) | 339.42 | 3.4 | 55.37 | 0.77 | HL1 |
| MOL001454 | [berberine](https://old.tcmsp-e.com/molecule.php?qn=1454) | 336.39 | 3.45 | 36.86 | 0.78 | HL2 |
| MOL002894 | [berberrubine](https://old.tcmsp-e.com/molecule.php?qn=2894) | 322.36 | 3.2 | 35.74 | 0.73 | HL3 |
| MOL002904 | [Berlambine](https://old.tcmsp-e.com/molecule.php?qn=2904) | 351.38 | 2.49 | 36.68 | 0.82 | HL4 |
| MOL001458 | [coptisine](https://old.tcmsp-e.com/molecule.php?qn=1458) | 320.34 | 3.25 | 30.67 | 0.86 | HL5 |
| MOL002907 | [CorchorosideA_qt](https://old.tcmsp-e.com/molecule.php?qn=2907) | 404.55 | 1.34 | 104.95 | 0.78 | HL6 |
| MOL002897 | [epiberberine](https://old.tcmsp-e.com/molecule.php?qn=2897) | 336.39 | 3.45 | 43.09 | 0.78 | HL7 |
| MOL000622 | [Magnograndiolide](https://old.tcmsp-e.com/molecule.php?qn=622) | 266.37 | 1.18 | 63.71 | 0.19 | HL8 |
| MOL008647 | [Moupinamide](https://old.tcmsp-e.com/molecule.php?qn=8647) | 313.38 | 2.86 | 86.71 | 0.26 | HL9 |
| MOL013352 | [Obacunone](https://old.tcmsp-e.com/molecule.php?qn=13352) | 454.56 | 2.68 | 43.29 | 0.77 | HL10 |
| MOL000785 | [palmatine](https://old.tcmsp-e.com/molecule.php?qn=785) | 352.44 | 3.65 | 64.6 | 0.65 | HL11 |
| MOL000762 | [PalmidinA](https://old.tcmsp-e.com/molecule.php?qn=762) | 510.52 | 4.52 | 35.36 | 0.65 | HL12 |
| MOL002668 | [Worenine](https://old.tcmsp-e.com/molecule.php?qn=2668) | 334.37 | 3.73 | 45.83 | 0.87 | HL14 |
